# Subtype-Conditioned NRF2–KEAP1 Signaling in Breast Cancer: A Narrative Review and Hypothesis-Generating Framework from Chemoprevention to Therapy Resistance

**DOI:** 10.3390/antiox15101223

**Published:** 2026-09-22

**Authors:** Hye Young Choi, Hayeong Kwon, Young-Sool Hah

**Affiliations:** 1 Department of Radiology, Gyeongsang National University Hospital and College of Medicine, 79 Gangnam-ro, Jinju 52727, Republic of Korea; whitecoc@hanmail.net; 2 Department of Radiology, Gyeongsang National University College of Medicine, 816-15 Jinju-daero, Jinju 52727, Republic of Korea; 3 Institute of Medical Science, Gyeongsang National University College of Medicine, 816-15 Jinju-daero, Jinju 52727, Republic of Korea; hykwon@gnu.ac.kr; 4 Biomedical Research Institute, Gyeongsang National University Hospital, 79 Gangnam-ro, Jinju 52727, Republic of Korea

**Keywords:** NRF2, KEAP1, breast cancer, triple-negative breast cancer, ferroptosis, redox addiction, biomarker, therapy resistance

## Abstract

NRF2 (nuclear factor erythroid 2-related factor 2) and its repressor KEAP1 (Kelch-like ECH-associated protein 1) govern the antioxidant response; their role in breast cancer is paradoxical: the same program that prevents malignant transformation can later drive survival and therapy resistance. We argue that this behavior is not a fixed switch but a continuum conditioned by molecular subtype, disease stage, and therapeutic context. Synthesizing primary mechanistic and clinical evidence, we find that NRF2 activation is predominantly non-mutational and that its pathological dependence is strongest not in triple-negative breast cancer (TNBC) as a whole but in a candidate redox-addicted subset—defined by nuclear NRF2 with reduced cytoplasmic KEAP1 protein, cystine/SLC7A11 reliance, and active ferroptosis defense. This subset is hypothesis-generating rather than validated: current support derives chiefly from a single tissue microarray cohort and cell-line studies, and the co-occurrence and prevalence of its features remain unestablished. NRF2-driven suppression of ferroptosis is one major, tractable mechanism of resistance among several. We frame these as directions for future research: no NRF2-directed intervention is ready for biomarker-guided selection, and systemic NRF2 inhibition risks on-target toxicity, because NRF2 also protects normal tissues from oxidative stress.

## 1. Introduction

Breast cancer is the most commonly diagnosed cancer among women worldwide [1] and comprises biologically distinct diseases. Its intrinsic molecular subtypes—luminal A, luminal B, human epidermal growth factor receptor 2 (HER2)-enriched, and basal-like—were established through landmark gene expression profiling [2,3]; triple-negative breast cancer (TNBC) is a receptor-defined clinical category enriched for, but not synonymous with, the basal-like subtype. These groups differ not only in their receptor status and clinical management [4,5] but also in their oxidative burden, metabolic wiring, and the selective pressures imposed by standard therapies [6].

Reactive oxygen species (ROS) play a dual role in biology: at controlled levels, they support proliferative signaling, whereas in excess, they damage DNA, lipids, and proteins and trigger cell death [7]. This duality can be exploited therapeutically because oncogenically transformed cells are selectively vulnerable to further increases in ROS [8]. The transcription factor nuclear factor erythroid 2-related factor 2 (NRF2; encoded by *NFE2L2*) is the master regulator of the inducible stress-response program that maintains ROS within tolerable limits by controlling antioxidant, detoxification, and metabolic genes through its repressor Kelch-like ECH-associated protein 1 (KEAP1).

The difficulty is that NRF2 is genuinely double-edged. The same pathway that prevents malignant transformation by detoxifying carcinogens and limiting oxidative damage in normal cells can, once a tumor is established, be co-opted to support tumor survival, metabolic adaptation, and therapy resistance. Several reviews have examined NRF2 signaling in cancer and in breast cancer broadly [9,10,11,12]. What remains insufficiently resolved is the biological context that determines which side of this duality predominates. In particular, few reviews have systematically organized NRF2 biology by breast cancer subtype, disease stage, ferroptosis vulnerability, and therapy-specific resistance mechanisms. The resulting literature affirms the importance of NRF2 without fully resolving the clinically relevant question: in which breast tumors, at which stage, and under which therapeutic conditions does NRF2 protect the host rather than the cancer?

This is a narrative, mechanistically oriented review that organizes primary and clinical evidence by breast cancer subtype, disease stage, treatment modality, and the strength of functional validation. We distinguish breast cancer-specific findings from evidence extrapolated from other NRF2/KEAP1-driven malignancies and explicitly label interpretations based on limited breast-specific data as hypothesis-generating.

### 1.1. Methods: Literature Search, Evidence Appraisal, and Levels of Inference

This narrative review used a structured but non-systematic literature search to identify evidence relevant to NRF2–KEAP1 signaling, redox metabolism, ferroptosis, and therapy resistance in breast cancer. PubMed was searched from database inception to 16 September 2026; Google Scholar was used for supplementary discovery, and ClinicalTrials.gov was consulted only to verify the registration and status of cited trials. Primary experimental, human tissue, cohort, and clinical studies were prioritized. Non-breast studies were retained only when they informed a mechanistic question not yet directly tested in breast cancer; such evidence is explicitly identified as extrapolated throughout. Full search terms, eligibility criteria, and the evidence appraisal procedure are provided in Supplementary Note S2.

Because this is a narrative rather than a systematic or scoping review, study identification, selection, and appraisal were performed by the authors and reconciled by consensus; duplicate independent screening was not undertaken and no protocol was prospectively registered. Evidence strength was evaluated using the rubric detailed in Supplementary Table S3, with greater weight given to human tissue-level and functional evidence than to transcript-only associations or single-model studies. The search was therefore designed to be reproducible and auditable, but not exhaustive.

To distinguish published evidence from interpretation, statements are classified as *demonstrated* (reported in human breast tumors or in breast cancer models with functional manipulation), *mechanistically supported* (following from evidence obtained in a related system, most often a non-breast *KEAP1*/*NFE2L2*-driven tumor, and not tested directly in breast cancer), or *proposed or testable* (an inference of ours, offered so that it can be refuted). The candidate redox-addicted TNBC subset, the composite NRF2-activation score, and predictions P1–P5 belong to the *proposed or testable* category. Accordingly, this review does not claim to establish a new breast cancer subtype, validate a biomarker, or provide evidence sufficient for treatment selection. For statements about therapy, we additionally record the source of the evidence on a four-tier scale, applied in the evidence tables of this review and in Supplementary Table S4: (i) direct breast cancer clinical evidence; (ii) breast cancer preclinical evidence; (iii) evidence obtained in other tumor types; and (iv) a conceptual proposal of the present authors. No therapeutic strategy tabulated in this review rests on tier (i), although tier (i) evidence now exists for the association between the NRF2 protein and chemotherapy response (Section 4.3 and Section 6.2).

### 1.2. Organizing Thesis, Terminology, and Relation to Previous Reviews

This review advances a single organizing thesis: the protective-versus-oncogenic behavior of NRF2 in breast cancer does not represent a fixed binary switch, but rather a continuum conditioned by molecular subtype, disease stage, and therapeutic context, and the strongest evidence for NRF2 as a therapeutic vulnerability lies in a candidate redox-addicted subset rather than in any molecular subtype as a whole. Throughout, we privilege primary mechanistic and clinical evidence and grade its strength, so that the subtype-conditioned account rests on what has been shown rather than on what is plausible.

*Terminology and scope.* By redox addiction, we mean an operationally defined functional state in which tumor survival or treatment resistance depends on sustained NRF2-driven antioxidant and ferroptosis-defense capacity—such that genetic or pharmacological disruption of NRF2 or downstream effectors, such as SLC7A11 or GPX4, impairs tumor survival or restores treatment sensitivity. The term, therefore, implies functional dependence rather than prognostic correlation alone.

We therefore reserve the term ‘dependency’ for functional evidence of this kind and use ‘association’ for prognostic or correlative findings. Because the available evidence is strongest in triple-negative disease, this review is necessarily TNBC-centered, whereas luminal and HER2-enriched contexts are treated as less-characterized secondary settings.

To make the novelty of this synthesis explicit rather than rhetorical, Table 1 compares the present review with major prior reviews of NRF2 biology and ferroptosis, including recent breast cancer- and TNBC-focused ferroptosis reviews [13]. These reviews provide important foundations but have not systematically integrated protein-level NRF2/KEAP1 stratification, ferroptosis-defense dependency, and biomarker-development logic in breast cancer. The present synthesis extends this literature by defining a candidate protein-level redox-addicted state characterized by nuclear NRF2 with reduced cytoplasmic KEAP1 protein expression, distinguishing breast cancer-specific from extrapolated evidence across the eight dimensions graded in this review and proposing a composite biomarker framework with a staged validation roadmap.

## 2. NRF2–KEAP1 Signaling Nodes Relevant to Breast Cancer Redox Adaptation

The molecular architecture of the NRF2–KEAP1 system has been reviewed exhaustively elsewhere, and the full pathway is summarized in Figure 1.

*Canonical degradation and its release.* NRF2 is a CNC-basic leucine zipper transcription factor [16] that heterodimerizes with small Maf proteins [17,18] to drive phase II detoxifying-enzyme expression through the antioxidant response element [19,20]; the axis has been comprehensively reviewed elsewhere [21,22,23]. Under basal conditions, KEAP1 holds NRF2 in the cytoplasm and, acting as the substrate adaptor for a Cullin-3–RBX1 E3 ubiquitin ligase, targets it for proteasomal destruction [24,25,26]; electrophilic modification of KEAP1 disrupts this interaction and prevents NRF2 degradation [27]. KEAP1 engages the Neh2 domain of NRF2 through high-affinity ETGE and lower-affinity DLG motifs that act as a “hinge-and-latch” that couples KEAP1 occupancy to NRF2 turnover [28]. Disruption of this engagement stabilizes NRF2 and permits its nuclear accumulation and ARE binding [29], a step that depends on its small Maf partners [30].

*Cysteine sensing.* Reactive cysteines on KEAP1—principally Cys151, Cys273, and Cys288—are the physiological switch: covalent modification by electrophiles or oxidants impairs KEAP1-dependent NRF2 degradation [31,32]; genetic substitution of these residues confirms their distinct, non-redundant roles in setting NRF2 activity in vivo [33,34].

*Non-canonical activation.* Several non-canonical mechanisms activate NRF2 independently of KEAP1 cysteine modification and are particularly relevant to breast cancer (Figure 1C): p62/SQSTM1-mediated sequestration of KEAP1 [35,36]; oncogene-driven upregulation of *NFE2L2* by KRAS, BRAF and MYC, required for tumorigenesis [37]; and Neh6-domain degradation via GSK-3β/β-TrCP–Cullin-1, which PI3K/AKT signaling, frequently hyperactive in breast cancer, disables, thereby stabilizing NRF2 without any mutation [38,39].

Notably, several of these non-canonical routes are documented specifically in breast cancer: oxidative-stress-induced binding of DPP3 to KEAP1 displaces NRF2 and supports breast cancer cell survival [40], while the DNA-repair proteins PALB2 and BRCA1 each engage the KEAP1–NRF2 axis—with PALB2 competing with NRF2 for KEAP1 binding to promote NRF2 accumulation [41] and BRCA1 interacting with NRF2 to sustain antioxidant signaling and cell survival [42]. Because NRF2 activation in breast cancer commonly arises through p62 accumulation, oncogenic signaling and PI3K/AKT activation, or metabolite-driven KEAP1 modification rather than fixed genomic lesions, mutational screening of *KEAP1*/*NFE2L2* is likely to miss a substantial proportion of NRF2-active breast tumors.

*Breast cancer-relevant outputs.* Genome-wide occupancy studies place several hundred genes under direct NRF2 control [43,44,45]. Regardless of how NRF2 is activated, the consequential outputs for this review are NRF2’s control of glutathione synthesis (glutamate–cysteine ligase catalytic and modifier subunits, GCLC and GCLM) [46], cystine import (solute carrier family 7 member 11, SLC7A11), the ferroptosis-limiting enzyme GPX4 and iron sequestration (FTH1), drug detoxification (NAD(P)H:quinone dehydrogenase 1, NQO1, and heme oxygenase 1, HMOX1) [47], and anabolic metabolic reprogramming [48,49,50]—an integration reviewed in detail elsewhere [51].

## 3. From Transient Cytoprotection to Persistent Tumor Fitness: The NRF2 Transition Point

*Transient activation as tissue protection.* In normal and preneoplastic cells, NRF2 activation is acute, reversible, and protective. By detoxifying carcinogens and electrophiles and maintaining redox and genomic integrity, transient NRF2 induction opposes cancer initiation—this is the rationale, examined in Section 7.1, for NRF2 activators as chemopreventive agents [52]. This protection is window-dependent, however: once transformation is underway, supplementing antioxidant capacity can instead accelerate tumor progression and metastasis in vivo [53,54].

*Persistent activation as a tumor-fitness program.* This relationship is inverted when NRF2 activation becomes constitutive. Sustained NRF2 activity furnishes a transformed cell with continuous antioxidant and detoxifying capacity, supports the metabolic demands of proliferation, and blunts the oxidative and lipid-peroxidative stresses that would otherwise limit growth or trigger death. That oncogene-driven NRF2 activation is required for KRAS-induced tumorigenesis indicates that, beyond a critical threshold, the antioxidant program is not incidental to malignancy but a mediator of it [37]; the same cytoprotective genes that shield normal cells then shield the tumor, conferring resistance to oxidative stress and chemotherapeutic insult—the so-called dark side of NRF2 [55], a role now integrated into accounts of how NRF2 supports multiple hallmarks of cancer [11] and of how a stress-protective pathway is co-opted during cancer evolution [56]. In breast cancer specifically, NRF2-driven engagement of the pentose phosphate pathway and a G6PD/HIF-1α/Notch1 axis couple this metabolic reprogramming to proliferation, migration, and epithelial-to-mesenchymal transition (EMT) [57], and NRF2 additionally promotes proliferation and metastasis through RhoA/ROCK signaling [58].

*What defines the transition?* The question that matters clinically is what tips protection into dependence. Duration and amplitude: a transient pulse protects, whereas chronic, high-amplitude activation drives persistent cellular reprogramming. Genotype: although *KEAP1*/*NFE2L2* mutations are uncommon in breast cancer, in squamous and lung carcinomas, loss-of-function KEAP1 or gain-of-function *NFE2L2* mutations lock the pathway into the persistent state [59,60,61,62]; more commonly in breast cancer, the persistent state is reached through the non-canonical routes described above—p62 accumulation, oncogenic signaling, or metabolite-driven KEAP1 modification. Microenvironment: hypoxia and the attendant ROS burden of the tumor core would be expected to favor sustained activation. Therapy pressure: critically, anticancer treatment is itself a driver of the transition—endocrine therapy can induce NRF2 in tumors that were initially NRF2-low, converting a protective pathway into a resistance mechanism under selection. This last point reframes the duality as partly iatrogenic: the transition is not only a property of the tumor’s natural history but something that treatment can precipitate.

Because the protective and pathological states are separated by conditions—duration, genotype, microenvironment, and therapy—rather than by an intrinsic property of “NRF2 in breast cancer,” the clinically actionable question becomes which tumors have crossed the transition into persistent NRF2 dependence.

## 4. Subtype-Conditioned NRF2 Biology in Breast Cancer

Breast cancer is not one disease but a collection of molecularly distinct entities—luminal A, luminal B, HER2-enriched, and basal-like—defined by intrinsic-subtype classifiers [63] and differing in their oxidative burden, their reliance on antioxidant programs, and the selective pressures imposed by their respective standard therapies (Figure 2) [6]. The receptor-defined triple-negative category, which we use throughout as the clinical entry point to basal-like biology, is enriched for but not synonymous with that subtype. The evidence for NRF2 as a therapeutic vulnerability, graded by subtype in Table 2, is strongest in a candidate redox-addicted subset rather than in TNBC as a whole.

### 4.1. Mapping NRF2/KEAP1 Status Across Molecular Subtypes

A first observation reframes how NRF2 activation should be understood in breast cancer. In lung, head-and-neck, and esophageal squamous carcinomas, constitutive NRF2 activity is driven predominantly by somatic loss-of-function mutations in *KEAP1* or gain-of-function mutations in *NFE2L2* that disrupt the ETGE/DLG degron interaction [74]; a pan-cancer catalog of somatic *NFE2L2* gain-of-function mutations likewise demonstrates that these alterations are concentrated in lung, bladder, uterine, and squamous tumors rather than in breast cancer [75]. Breast cancer does not follow this pattern. In the original Cancer Genome Atlas analysis of primary breast tumors, only three genes—*TP53*, *PIK3CA*, and *GATA3*—were mutated at frequencies exceeding 10% across all cases, and neither *NFE2L2* nor *KEAP1* reached this threshold in any subtype [6]. The implication is mechanistically important: where NRF2 is activated in breast cancer, that activation is likely to arise largely through non-mutational mechanisms. This distinction has important implications for both biomarker development (Section 4.5) and therapeutic strategy (Section 7) because tumors lacking *KEAP1*/*NFE2L2* mutations may nonetheless be functionally NRF2-high. Three states that the literature often treats as interchangeable are kept separate throughout this review: reduced or absent cytoplasmic KEAP1 protein, assessed by immunohistochemistry; functional KEAP1 inactivation, arising from cysteine modification, p62-dependent sequestration, or metabolite-driven cysteinylation without any change in protein level; and genomic KEAP1 alteration, which is uncommon in breast cancer. The candidate phenotype proposed here is defined at the protein level only.

A second observation concerns the discordance between transcript and protein expression. Reduced *NFE2L2* mRNA has been associated with poorer outcomes in some breast cancer cohorts [76], whereas elevated nuclear NRF2 protein—and germline variation in *NFE2L2* and *KEAP1*—has been associated with disease progression, chemoresistance, and poorer survival [65,77,78]. Bulk transcriptomic measurement of *NFE2L2* is therefore an unreliable surrogate for NRF2-pathway activity, a caveat that recurs throughout the subtype literature and to which we return in Section 4.5. Throughout this section, we prioritize protein-level and functional evidence over mRNA-only correlations, and grade each line of evidence accordingly in Table 2.

### 4.2. Luminal (ER-Positive) Breast Cancer

Luminal tumors, which express the estrogen receptor (ER) and depend on endocrine signaling, carry a comparatively low constitutive oxidative burden and show the weakest baseline dependence on NRF2 among the major subtypes. The evidence here is best described as moderate and mechanistic rather than clinical.

Where NRF2 is engaged in luminal cells, it intersects with the metabolic and proliferative machinery characteristic of this subtype. In MCF-7 cells (a luminal model), forced NRF2 overexpression—or KEAP1 knockdown—upregulates glucose-6-phosphate dehydrogenase (G6PD) and transketolase, the rate-controlling enzymes of the pentose phosphate pathway, and drives a G6PD/HIF-1α/Notch1 axis that promotes proliferation, migration, and EMT; the converse manipulations (NRF2 knockdown, KEAP1 overexpression) reverse these effects [57]. A parallel mechanism connects NRF2 to the motility machinery: in the same MCF-7 and MDA-MB-231 models, NRF2 sustains RhoA/ROCK signaling—acting as a transcriptional silencer of the estrogen-related receptor-α promoter to stabilize RhoA—so that NRF2 silencing suppresses proliferation, migration, and metastasis [58]. Notably, the study supporting the NRF2–PPP connection used both MCF-7 and the basal MDA-MB-231 line and observed concordant effects, indicating that this metabolic mechanism is not subtype-exclusive but is superimposed on subtype-specific baseline metabolism [57].

The more clinically consequential role of NRF2 in luminal disease is in endocrine-therapy resistance, which is discussed in detail in Section 6.1. In brief, the relevant feature of luminal biology is that NRF2 activation appears to be an acquired, therapy-induced event—emerging under the selective pressure of tamoxifen—rather than a constitutive property of the untreated tumor. This temporal pattern is consistent with the transition-point framework developed in Section 3: in luminal disease, persistent NRF2 activation is something the tumor acquires, not something it begins with.

### 4.3. HER2-Enriched Breast Cancer

The HER2-enriched subtype occupies an intermediate and understudied position. Two features of its biology make NRF2 engagement plausible. First, HER2-enriched tumors carry the highest somatic mutation burden of any breast cancer subtype, with *TP53* mutated in roughly 72% and *PIK3CA* in roughly 39% of cases [6]; the high prevalence of PI3K pathway activation provides a route to oncogene-driven NRF2 induction, independent of KEAP1 status. Second, HER2 (ERBB2) signaling is expected to generate ROS and engage antioxidant adaptation, creating a context in which NRF2 activity would support tumor survival; however, this sequence has not been demonstrated directly in HER2-enriched breast tumors.

Direct, subtype-resolved evidence for NRF2 dependence in HER2-enriched disease is nonetheless limited, and we grade it as low. The clearest functional evidence is pharmacological: in HER2-positive breast cancer cells, NRF2 inhibition with brusatol represses the NRF2/HO-1 and HER2–AKT/ERK1/2 axes and synergizes with trastuzumab, reducing tumor burden in xenografts [69]. Patient-level, HER2-resolved NRF2 data have now begun to appear. In paired pre- and post-chemotherapy biopsies from 75 patients with HER2-positive and 56 with triple-negative breast cancer, NRF2 protein was scored separately in tumor and in stroma, and its upregulation, together with that of the peroxiporin AQP5, was associated with failure to achieve a pathological complete response [79]. This is a single retrospective cohort reporting a surrogate endpoint, and models of acquired anti-HER2 resistance that directly interrogate NRF2 are still lacking; but the HER2-enriched column of Table 2 is no longer empty of direct human evidence. The most defensible statement is therefore that NRF2 is likely relevant to anti-HER2 therapy resistance through redox buffering of HER2-driven oxidative stress—supported by the brusatol–trastuzumab synergy above and by the high prevalence of PI3K-pathway activation that can drive NRF2—but, absent dedicated HER2-subtype clinical studies, this proposition rests more on convergent pathway logic than on subtype-specific human evidence. Because brusatol is not NRF2-selective, this evidence should be interpreted as pharmacological proof of concept rather than as definitive evidence of HER2-specific NRF2 dependency. We explicitly flag this as a gap rather than present indirect evidence as if it were subtype-specific.

### 4.4. Triple-Negative Breast Cancer and Candidate NRF2-High Redox-Addicted States

Triple-negative breast cancer (TNBC) is where the case for NRF2 as a determinant of outcome and a therapeutic target is strongest—but the evidence requires a precise rather than a categorical reading. TNBC accounts for approximately 15–20% of breast cancers and lacks ER, progesterone receptor, and HER2 expression, leaving cytotoxic chemotherapy as the mainstay of systemic treatment and conferring high recurrence rates and poor prognosis [6]. The therapeutic landscape is now biomarker-driven: immune-checkpoint blockade in early TNBC [80] and in PD-L1-positive metastatic disease [81], PARP inhibition in germline BRCA1/2-mutated disease [82], TROP2-directed antibody–drug conjugates [83], and first-line sacituzumab govitecan with pembrolizumab [84], all from randomized phase III trials [80,81,82,83,84], with first-line approval of sacituzumab govitecan in 2026 [84,85,86]. The contemporary TNBC backbone is therefore biomarker-defined. It is also internally heterogeneous, comprising basal-like, mesenchymal, mesenchymal stem-like (MSL), immunomodulatory, and luminal-androgen-receptor states [87,88], a structure foreshadowed by earlier mesenchymal and claudin-low classifications [89]. This internal structure is central to the argument of this review: the NRF2-dependent phenotype is not uniform across TNBC but is concentrated in a candidate redox-addicted subset.

The clearest prognostic evidence comes from a tissue microarray study of 203 patients with operable TNBC, in which NRF2 and KEAP1 proteins were scored by immunohistochemistry and dichotomized at the median H-score [65]. The combination carried strong prognostic information: patients with high nuclear NRF2 and low cytoplasmic KEAP1 had markedly worse outcomes (5-year overall survival 70.4%; disease-free survival 63.0%) than those with low NRF2 and high KEAP1 (overall survival 96.8%; disease-free survival 95.2%), with intermediate groups falling between (overall survival *p* < 0.001; disease-free survival *p* = 0.001) [65]. Two points deserve emphasis. First, it was the protein-level combination—nuclear NRF2 together with loss of its cytoplasmic inhibitor—that stratified patients, vindicating the multi-parameter approach advocated in Section 4.5. Second, the worst-prognosis group represented a subset of TNBC, not the entire TNBC population, consistent with the candidate redox-addicted-subset framing. Two caveats temper this anchor, however: it derives from a single-institution retrospective cohort, and the use of the median H-score as the dichotomization threshold is data-derived rather than prespecified, which may overstate prognostic separation and limit its generalizability as a transferable clinical threshold.

The mechanistic basis for this addiction has been defined most sharply in the MSL subtype. Bottoni and colleagues showed that MSL TNBC cells, which overexpress the cystine/glutamate antiporter SLC7A11/xCT—itself identified as a common therapeutic dependency of triple-negative tumors [73]—and take up large amounts of exogenous cystine, use that cystine to activate NRF2 through direct cysteinylation of KEAP1—a covalent modification of the inhibitor rather than a mutation [66]. Critically, the resulting protection against oxidative stress operated through a glutathione-independent arm: NRF2 induced a transcriptional program (including OSGIN1, RGS17, SRXN1, and AKR1B10, themselves negative prognostic markers in TNBC) that defended the cells against cystine-deprivation death, and re-expression of OSGIN1 alone could substitute for NRF2 in preventing this phenotype [66]. This identifies a cystine/NRF2/OSGIN1 survival axis specific to a candidate subset of redox-addicted TNBC. The dependence cuts both ways: when cystine is withheld, TNBC cells are correspondingly susceptible to death, with cystine starvation activating a GCN2–eIF2α–ATF4 program that induces CHAC1-mediated glutathione degradation and drives necroptosis and ferroptosis [90]—the vulnerability that the cystine/NRF2 survival axis exists to counter. In TNBC, SLC7A11 is further stabilized at the membrane by a CD44-variant–MUC1-C complex that sustains glutathione and resists erastin-induced ferroptosis [91]. Consistent with broader remodeling of cysteine metabolism in basal-like breast cancer, basal-like breast tumors reprogram the transsulfuration pathway and realign cellular cysteine persulfidation to support progression [92], reinforcing the view that cystine/cysteine handling is a defining axis of the candidate redox-addicted state rather than an incidental feature.

Beyond this subset, NRF2 contributes to the metabolic phenotype of TNBC more broadly: NRF2 silencing suppresses HIF-1α accumulation and lowers glycolytic enzyme expression, linking NRF2 to glycolytic reprogramming in breast cancer [57], and NADPH-oxidase–coupled redox signaling downstream of NRF2 supports the mesenchymal, migratory phenotype through stabilization of SNAIL [93]. The cumulative picture is of a subtype in which a candidate subset of tumors may be genuinely dependent on NRF2-mediated redox buffering—and in which that fraction can, in principle, be identified by the protein and functional markers described above—rather than a subtype uniformly characterized by NRF2 dependence. Critically, this subset remains a hypothesized convergence rather than an established entity: its defining protein, metabolic, and lipid features are drawn from different datasets and models, their co-segregation within individual tumors has not been directly demonstrated, and the proportion of TNBC meeting all criteria is unknown—gaps that make it a construct to be validated prospectively, not a characterized population. Conflating the subset with the category risks limiting the translational value of NRF2 inhibition.

### 4.5. Evidence Caveats and the Biomarker Problem

The subtype analysis above is only as reliable as the measurements that underpin it, and four measurement problems warrant explicit statement.

First, *NFE2L2* mRNA is a poor proxy for NRF2 activity. Because NRF2 activation in breast cancer frequently occurs through post-translational mechanisms—including KEAP1 cysteinylation, p62-dependent KEAP1 sequestration, and oncogene-driven stabilization—transcript abundance can be uninformative or even inversely related to pathway output [65,66]; illustrating this discordance, reduced rather than elevated *NFE2L2* mRNA has been associated with poor outcomes in independent breast cancer cohorts (METABRIC and a 176-tumor qRT-PCR set) [76], whereas poor prognosis has been linked to high NRF2 protein in independent analyses, including a meta-analysis in which NRF2 overexpression predicted worse overall and disease-free survival (pooled hazard ratios 1.82 and 1.79) [94]. This discordance suggests that protein-level and functional readouts may better reflect pathologically active NRF2 signaling than transcript abundance alone. Prognostic stratification has been demonstrated using the combination of nuclear NRF2 and KEAP1 protein expression [65], while the value of nuclear NRF2 immunolocalization as a prognostic readout in breast cancer was reported in earlier patient cohorts [95].

Second, antibody specificity for NRF2 immunohistochemistry has been a recurring concern, and discordant antibody performance contributes to the heterogeneous expression estimates across cohorts. Functional readouts—an NRF2 target-gene signature or the OSGIN1/SRXN1-type output identified in cystine-dependent MSL TNBC cells—provide a more robust estimate of activity than staining intensity alone [66].

Third, bulk tumor profiling conflates malignant and stromal compartments. Because NRF2 is active in fibroblasts, immune cells, and the endothelium as well as in cancer cells, a bulk NRF2 signature may reflect the microenvironment rather than the tumor cell compartment. Single-cell and spatial approaches are needed to attribute NRF2 activity to specific cell populations, particularly when the biomarker is intended to guide cell-autonomous targeting.

Fourth, NRF2 activity is not uniform within a single tumor, so that even a compartment-resolved measurement may be regionally misleading. The determinants invoked throughout this review—hypoxia, reactive oxygen species burden, cystine and nutrient availability, and prior therapy exposure—are themselves spatially organized, each being most extreme in the poorly perfused tumor core and least so at the invasive margin. Direct evidence that the NRF2-activation state tracks this geography is now available: spatial transcriptomic profiling across murine and human tumors resolves a gradient in which pro-inflammatory CXCL9-positive macrophages occupy the tumor periphery while NRF2-imprinted SPP1-positive macrophages accumulate around necrotic regions [96]. That observation was made in the myeloid compartment, but the stressors that drive it act on malignant cells in the same spatial pattern, and these shared spatial stressors provide a rationale to test whether malignant-cell NRF2 activity is similarly heterogeneous. A tumor scored NRF2-high on a single block may therefore contain regions in which the pathway is quiescent, and a tumor scored NRF2-low may contain a focal NRF2-high population that could be missed by single-region sampling. The practical consequence is that the reproducibility of any NRF2-activation score will depend as much on the sampling strategy as on the antibody performance or scoring threshold, and agreement studies using one block per tumor will overstate reproducibility. We therefore treat multi-region sampling or a spatially resolved readout as a minimum requirement for prospective evaluation and single-region immunohistochemistry as insufficient for the compartment-resolution component of the framework proposed in Section 8.3; the supporting evidence is set out in Supplementary Note S1.

These caveats motivate the conceptual proposal we develop in Section 8: that “NRF2-high breast cancer”, if it is to function as a treatment-selection criterion, should be defined by a composite of protein-level, transcriptional, and functional readouts rather than by any single marker.

## 5. NRF2, Ferroptosis Defense, and Lipid Peroxide Control

Ferroptosis defense is one of NRF2’s outputs, among several, and the emphasis placed on it here reflects therapeutic tractability rather than mechanistic primacy. The same transcriptional program supplies NADPH through the pentose phosphate pathway, supports nucleotide and serine biosynthesis, sustains glutamine utilization, induces xenobiotic-detoxifying and efflux machinery, and contributes to proteostasis and DNA-repair capacity; each can confer therapy resistance independently of lipid peroxide control. Several of these parallel outputs have direct breast cancer support: pentose phosphate pathway activation through G6PD and transketolase [57], induction of multidrug-efflux transporters and NQO1 under tamoxifen [97], glutathione synthesis through GCLC and GCLM under hypoxia [72], transcriptional support of homologous recombination through RAD51 [98], reprogramming of transsulfuration and cysteine persulfidation in basal-like tumors [92], and maintenance of a stem-like state [66,99]. Any of these can generate an NRF2-associated resistance phenotype without lipid peroxidation being involved, and a study that infers ferroptosis defense from NRF2 status alone does not discriminate among them. Ferroptosis is foregrounded because it is a pharmacologically addressable convergence point at which NRF2-dependent survival and treatment sensitivity can be interrogated. Where a resistance phenotype has been attributed to NRF2 without distinguishing among these outputs, we explicitly state that limitation. This section, therefore, examines the suppression of ferroptosis as a principal mechanism by which NRF2 dependency may be exerted in the tumors identified above (Figure 3). Ferroptosis is an iron-dependent form of regulated cell death driven by the accumulation of phospholipid hydroperoxides in cellular membranes, mechanistically and morphologically distinct from apoptosis [100,101,102,103].

### 5.1. NRF2 as a Central Transcriptional Regulator of Ferroptosis Defense

The core anti-ferroptotic axis is the system xc^−^/glutathione/GPX4 pathway, and NRF2 governs it at multiple nodes. System xc^−^, whose functional subunit is SLC7A11, imports cystine in exchange for glutamate; cystine is reduced to cysteine and used to synthesize glutathione, the essential cofactor for GPX4 [100]. GPX4 is the only enzyme capable of directly reducing complex phospholipid hydroperoxides to non-toxic alcohols, and its genetic or pharmacological inactivation is sufficient to trigger ferroptosis [104,105]. SLC7A11 is a direct NRF2 target [106,107,108], and NRF2 additionally controls the genes governing glutathione synthesis (GCLC, GCLM, GSS), iron sequestration (the ferritin subunit FTH1)—counterbalancing the transferrin-mediated iron supply that fuels peroxidation [109,110]—and NADPH-regenerating and detoxifying enzymes (NQO1, the heme-catabolizing HMOX1), such that NRF2 status sets the overall ferroptotic threshold of the cell [14]. Beyond the GSH/GPX4 arm, parallel GPX4-independent systems—ferroptosis suppressor protein 1 (FSP1), which regenerates reduced coenzyme Q10; GTP cyclohydrolase 1 (GCH1), which supplies the radical-trapping antioxidant tetrahydrobiopterin (BH4); and mitochondrial dihydroorotate dehydrogenase (DHODH)—provide redundant protection against membrane lipid peroxidation [111,112,113,114], a redundancy that limits how completely ferroptosis can be forced. The resulting vulnerability of therapy-resistant, mesenchymal-state cancer cells to ferroptosis, and its therapeutic exploitation, have been extensively reviewed [115,116].

The most direct demonstration that NRF2 status determines ferroptotic fate in cancer came from hepatocellular carcinoma, where Sun and colleagues showed that p62-dependent sequestration of KEAP1 stabilized NRF2 and, through transcriptional upregulation of NQO1, HMOX1, and FTH1, protected cells against ferroptosis induced by erastin and sorafenib; knockdown of NRF2 or of these individual targets restored ferroptotic sensitivity and enhanced the anti-tumor activity of these agents both in vitro and in xenografts [117]. The principle supported there—that NRF2 activity is an important determinant of the therapeutic response to ferroptosis-inducing treatment—may extend to breast cancer, with the important qualifier (Section 4.4) that the NRF2-defended state in TNBC may operate partly through glutathione-independent outputs such as OSGIN1 rather than through GSH/GPX4 alone [66].

### 5.2. The Redox–Lipid Interface: Fatty-Acid Handling as a Conditioning Layer

Ferroptosis is not determined by antioxidant capacity alone; it is equally a function of the substrate available for peroxidation. Polyunsaturated fatty acids (PUFAs) esterified into membrane phospholipids are the proximal targets of lipid peroxidation, and the enzymatic machinery that loads PUFAs into membranes therefore conditions ferroptotic sensitivity in a manner orthogonal to NRF2’s antioxidant output. The rate-defining step is catalyzed by acyl-CoA synthetase long-chain family member 4 (ACSL4), which esterifies long ω6 PUFAs—chiefly arachidonic and adrenic acids—into phosphatidylethanolamine, generating the peroxidation-prone lipids whose oxidation—specifically of arachidonoyl- and adrenoyl-phosphatidylethanolamines—executes ferroptosis [67,118,119]. The relevance of this enzyme to breast cancer is direct and subtype-specific: in a genome-wide CRISPR screen and lipidomic analysis, Doll and colleagues found that ACSL4 was preferentially expressed across a panel of basal-like breast cancer cell lines, and its expression predicted ferroptotic sensitivity [67]. This places PUFA-handling capacity alongside NRF2 status as a co-determinant of whether a basal-like/TNBC cell can be driven into ferroptosis, and it provides a second, lipid-side axis that may help define the candidate redox-addicted subset described in Section 4.4.

Fatty-acid-binding proteins (FABPs)—small intracellular lipid chaperones that traffic fatty acids to organelles and transcriptional machinery—represent one illustrative node at this redox–lipid interface and one with emerging breast cancer data. FABP5 is overexpressed in breast cancer in proportion to malignancy—correlating with the triple-negative phenotype, high histological grade, and worse disease-free survival, with reduced tumor growth and lung metastasis in FABP5-null hosts [120]—and, with FABP7, participates in PUFA transport in TNBC cells [121]. Pharmacological inhibition of FABP5 with SBFI26 induced ferroptosis preferentially in MDA-MB-231 (basal/TNBC) cells, raising malondialdehyde, iron, and oxidized glutathione while depleting reduced glutathione; the effect was rescued by the ferroptosis inhibitor ferrostatin-1 and by glutathione but not by erastin, and transcriptomic profiling showed downregulation of *NFE2L2* alongside upregulation of pro-ferroptotic effectors (HMOX1, ALOX12, ALOX15, SAT1, CHAC1) [121]. The mechanistic interpretation most consistent with these data is that disrupting FABP-mediated lipid trafficking shifts the membrane lipid environment and the NRF2 balance toward ferroptotic vulnerability—positioning FABPs as a conditioning rather than a master variable. We deliberately keep this to a single node: the FABP literature is largely correlative with respect to ferroptosis, and overstating it would distort the NRF2-centered account. More broadly, the desaturation machinery that builds PUFAs—FADS1 and FADS2—has been shown to control lipid composition and ferroptosis susceptibility specifically in TNBC, reinforcing the general point that lipid-metabolic wiring sets the stage for NRF2’s antioxidant program [122].

### 5.3. Ferroptosis Vulnerability as a TNBC-Selective Therapeutic Opportunity

In the model proposed here, a candidate redox-addicted TNBC cell is one that would combine high reliance on NRF2-mediated peroxide defense (Section 4.4) with a basal-like lipid program that loads membranes with peroxidation-prone PUFAs (Section 5.2), making it simultaneously dependent on ferroptosis defense and vulnerable to its disruption. Whether these two properties co-occur in identifiable patient tumors has not been demonstrated; the formulation is therefore a testable model rather than a described cell type. Wild-type p53 can repress SLC7A11 transcription, thereby reducing cystine uptake and sensitizing cells to ferroptosis [123]. Loss of p53 function may therefore converge with NRF2-driven SLC7A11 upregulation to reinforce ferroptosis resistance. This dual character explains why TNBC, and the basal-like subset in particular, is repeatedly nominated as the breast cancer entity most amenable to ferroptosis-inducing therapy.

The same axis, left intact, is also a mechanism of acquired drug resistance. In breast cancer cells, miR-141-3p—elevated and associated with poor prognosis—directly represses KEAP1 (validated by luciferase reporter assay), thereby de-repressing NRF2 and its downstream SLC7A11–GSH–GPX4 program; the consequence is attenuated ferroptosis and resistance to both paclitaxel and the GPX4 inhibitor RSL3, an effect reversed by the NRF2 inhibitor ML385 and recapitulated in vivo [70]. This illustrates the central logic of this review: the very NRF2 activity that protects the candidate redox-addicted cell against oxidative stress is what allows it to evade therapies whose efficacy depends, at least in part, on lipid peroxidation. It also identifies the actionable corollary—that pharmacological restoration of ferroptotic sensitivity (by inhibiting NRF2, SLC7A11, or GPX4) is a route to resensitizing resistant TNBC, a strategy we evaluate critically in Section 7.

Two cautions temper this opportunity. First, ferroptotic sensitivity is heterogeneous within TNBC, reflecting the lipid and redox features above rather than the triple-negative immunophenotype per se; the target population is again the candidate redox-addicted subset, not the category. Second, because NRF2’s protective output in some TNBC cells runs through glutathione-independent effectors [66], strategies aimed solely at the GSH/GPX4 arm may be incompletely effective in precisely the cells that are most NRF2-addicted—an argument for combination or upstream targeting, which Section 7 develops.

## 6. NRF2-Driven Resistance Across Treatment Modalities

The mechanisms discussed above converge on a clinically important consequence: NRF2 activity not only marks aggressive breast cancer but can also actively protect tumors against therapeutic stress and contribute to treatment resistance. The modality determines both the mechanism of resistance and the point at which NRF2 inhibition might restore sensitivity. A unifying theme across these treatment modalities is that oxidative stress contributes, directly or indirectly, to therapeutic response, whereas NRF2-mediated redox adaptation can blunt this vulnerability. More broadly, much of the mechanistic framework in this and the following sections is informed by studies in non-breast *KEAP1/NFE2L2*-driven tumors—including p62–KEAP1-mediated ferroptosis defense in hepatocellular carcinoma, NQO1–ferroptosis–immunity coupling in KEAP1-mutant (largely non-breast) tumors, and immune-evasion and glutaminase-dependence mechanisms in lung adenocarcinoma. Accordingly, where these mechanisms have not yet been demonstrated directly in breast cancer, we present them as biologically informed, hypothesis-generating models rather than established breast cancer-specific mechanisms.

### 6.1. Endocrine-Therapy Resistance in ER-Positive Disease

Endocrine therapy is the backbone of treatment for the roughly 80% of breast cancers expressing ERα, yet a substantial proportion of treated patients develop resistance and recurrence. The most instructive evidence that NRF2 mediates this resistance—and that the resistance is therapy-induced rather than pre-existing—comes from a study combining cell models with paired patient biopsies [64]. Tamoxifen-resistant (TAM-R) derivatives of MCF7 and MDA-MB-231 cells displayed lower ROS levels, higher antioxidant capacity, and elevated NRF2; in addition, MCF7 TAM-R cells exhibited a shift toward glycolysis, and NRF2 inhibition re-sensitized both models to irradiation [64]. In tumor tissue from patients treated with short-term presurgical tamoxifen (NCT00738777), NRF2 protein—undetectable at baseline—rose to positivity in 50–90% of tumor cells after treatment (*p* = 0.0008), whereas paired patients treated with anastrozole ± fulvestrant from the same trial showed no comparable induction (0–10% NRF2-positive cells) [64]. Independent work reinforces the mechanism: tamoxifen itself drives an oxidative, NRF2-dependent transcriptional response that upregulates antioxidant genes and multidrug-efflux transporters (ABCC1, ABCC3, NQO1), with high tumoral NRF2/ABCC1/ABCC3/NQO1 predicting worse survival after tamoxifen [97]; and a splice variant of the co-repressor NCOR2 (BQ323636.1) confers tamoxifen and epirubicin resistance by augmenting NRF2 transcriptional activity in patient tumors [124]. It also reveals an important cross-modality consequence: TAM-R cells are cross-resistant to irradiation via the same NRF2-dependent antioxidant program, linking endocrine resistance to radioresistance through NRF2.

### 6.2. Chemotherapy Resistance Under Oxidative and Hypoxic Stress

For the candidate redox-addicted TNBC subset, which relies on cytotoxic chemotherapy rather than targeted endocrine or HER2 agents, resistance is shaped by the same stresses that make these tumors NRF2-dependent: many chemotherapeutics kill in part by generating ROS, and the poorly vascularized, hypoxic tumor core raises ROS further, so NRF2-driven antioxidant and detoxification programs become a recurrent route to chemoresistance. In MCF7 cells, hypoxia increased ROS and NRF2 activity and induced the glutathione-synthetic enzymes GCLC and GCLM; NRF2 overexpression conferred chemoresistance even under normoxia, and—critically for the therapeutic framework developed in this review—chemical inhibition of NRF2 increased cisplatin cytotoxicity in an in vivo model, defining a ROS–NRF2–GCLC–GSH axis as a driver of hypoxia-associated drug resistance [72]. Beyond glutathione synthesis, NRF2 controls drug-detoxifying and efflux machinery, and silencing NRF2 increases ROS and re-sensitizes cancer cells to chemotherapy in vivo [125]. Within this subset specifically, the antioxidant program is further reinforced by the cystine/NRF2 axis, which adds a partly glutathione-independent layer of protection against oxidative chemotherapeutic stress [66]. The ferroptosis-evasion arm of chemoresistance—exemplified by miR-141-3p-driven paclitaxel resistance—is treated separately in Section 6.4 because it operates across modalities. In human tumors, the same direction of effect has now been observed: NRF2 protein rose after neoadjuvant chemotherapy in paired biopsies, and higher NRF2 in both the tumor and the surrounding stroma was associated with failure to achieve a pathological complete response [79]; this is the first direct clinical evidence in breast cancer that NRF2 status tracks chemotherapy response, and, because the two compartments were scored separately, the first to indicate that part of the signal is stromal (Section 6.5).

### 6.3. Radioresistance and Redox Buffering

Ionizing radiation kills tumor cells substantially through ROS-mediated and direct DNA damage, so the antioxidant capacity that NRF2 confers is intrinsically radioprotective—a benefit to normal tissue but a liability when it shields the tumor—and the gain of NRF2 function is sufficient to confer radioresistance in cancer cells [126]; the interplay of ferroptosis, radiotherapy, and NRF2/SLC7A11-mediated radioresistance has been reviewed in detail [127]. Two breast cancer-relevant mechanisms are now defined. First, NRF2 buffers radiation-induced ROS: in TNBC cells, irradiation upregulated NRF2 and drove its nuclear translocation in a ROS-dependent manner, and NRF2 overexpression attenuated—while knockdown potentiated—radiation killing [71]. Second, and less expected, NRF2 promotes DNA repair independently of its antioxidant role: studies in MCF7 breast cancer cells and A549 lung cancer cells showed that NRF2 inhibition slowed repair of radiation-induced DNA damage and reduced RAD51 expression, impairing homologous recombination, with residual damage persisting even in the presence of the ROS scavenger N-acetylcysteine [98]. The mechanistic implication is that NRF2 supports radioresistance through at least two separable channels—redox buffering and transcriptional support of DNA repair—so that radiosensitization by NRF2 inhibition need not depend solely on increasing ROS levels.

### 6.4. Ferroptosis Evasion as a Cross-Therapy Survival Mechanism

NRF2 suppresses ferroptosis; the point here is that ferroptosis evasion provides a therapeutically actionable mechanism that can contribute to resistance across several treatment modalities, which is why it merits its own heading. Because paclitaxel, certain platinum agents, and radiotherapy can each engage lipid peroxidation as part of their cytotoxic activity, an NRF2-high ferroptosis-defended state confers protection that is not modality-specific. The clearest evidence for breast cancer is the miR-141-3p axis: by directly repressing KEAP1 and thereby activating the NRF2–SLC7A11–GSH–GPX4 program, miR-141-3p attenuated ferroptosis and conferred resistance to both paclitaxel and the GPX4 inhibitor RSL3, effects that are reversible by the NRF2 inhibitor ML385 and reproduced in vivo [70]. The cancer-stem-cell compartment exemplifies the same logic in the radiation setting: NRF2 inhibition with ML385 sensitized TNBC stem cells (CSCs) to irradiation, linking the self-renewing, treatment-refractory population directly to NRF2-dependent survival [71], consistent with the NRF2-dependent radioresistance of ALDH-high breast cancer stem cells [99]. Stemness itself is reinforced by NRF2: in the cystine-addicted TNBC cells discussed above, NRF2 induces a stem-like transcriptional program, linking the antioxidant response to the cellular reservoir implicated in relapse [66]. The therapeutic corollary—pharmacological restoration of ferroptotic sensitivity as a strategy to overcome multimodal resistance—is developed in Section 7.

### 6.5. Tumor Immune Microenvironment and Immunotherapy Resistance

Immune-checkpoint blockade has become part of the treatment landscape for TNBC, making the relationship between NRF2 and anti-tumor immunity directly relevant. One breast cancer-relevant link has nonetheless been reported: in recurrent TNBC, ferroptosis inducers upregulate PD-L1 (CD274), and co-expression of CD274 with ferroptosis drivers marks tumors at a higher risk of recurrence, suggesting a potential synergy between ferroptosis induction and PD-L1 blockade [128]. A mechanistic basis for coupling ferroptosis to anti-tumor immunity has been demonstrated: immunotherapy-activated CD8^+^ T cells release interferon-γ, which represses the system xc^−^ subunits SLC7A11 and SLC3A2, thereby restraining tumor cystine uptake and promoting lipid peroxidation and ferroptosis as a mode of CD8-mediated killing [129]. Because NRF2 transcriptionally drives SLC7A11, an NRF2-high, SLC7A11-high state would be predicted to oppose precisely this axis—directly connecting the candidate redox-addicted phenotype of Section 4 and Section 5 to a plausible mechanism of immunotherapy resistance. Across multiple cancers, constitutive NRF2 activation is associated with impaired responses to anti-PD-1 therapy, with mechanistic support for immune evasion from engineered models, in vitro studies, and clinical trial samples [130]. The most direct causal demonstration comes from lung adenocarcinoma, where KEAP1-mutant tumors harbor reduced immune-cell infiltration and a less active immune microenvironment and resist checkpoint blockade despite a high tumor mutational burden [131]. Whether this circuitry operates equivalently in breast cancer has not been established, and we present it as a mechanistically plausible extrapolation requiring breast-specific validation rather than as demonstrated breast cancer biology. To be explicit about the evidence level, the ferroptosis–PD-L1 link is breast cancer-specific but preclinical [128]; the immune-evasion mechanism is TNBC-plausible but demonstrated chiefly in lung adenocarcinoma [130,131]; and the resulting combination strategy should therefore be regarded as a testable translational hypothesis rather than an established therapeutic approach. The therapeutic hypothesis, which motivates combining NRF2-pathway inhibition or ferroptosis induction with checkpoint blockade in the candidate redox-addicted TNBC subset, is taken up as a forward-looking strategy in Section 7 and Section 8, with the caveat that NRF2 also operates in normal immune cells, so systemic inhibition risks on-target immune toxicity.

Breast cancer-specific evidence now indicates that host NRF2 can alter tumor behavior across several compartments. In breast patient-derived cancer-associated fibroblasts, NOX4-driven NRF2 activation supported fibroblast survival and autophagy, while stromal NOX4 deletion or inhibition reduced orthotopic mammary tumor growth and metastasis [132]. In an orthotopic luminal-B breast cancer model, host *Nrf2* deficiency altered both the tumor redox profile and antioxidant/NADPH programs in cancer-associated adipose tissue, supporting bidirectional redox coupling between the tumor and surrounding adipose tissue [133]. This has since been extended to brown adipose tissue: in an orthotopic breast cancer model, tumor growth reprogrammed the redox and metabolic profile of brown adipose tissue in an *Nrf2*-dependent manner, with advanced tumors downregulating glycolytic and pentose phosphate pathway proteins, so that host *Nrf2* shapes systemic as well as local metabolic adaptation [134]. A 2025 study further showed that constitutive hematopoietic NRF2 activation accelerated therapy-naive MMTV-PyMT mammary tumor growth; in separate immunotherapy models, macrophage-specific *Nrf2* deletion enhanced anti-CD40 and anti-PD-1 responses, and spatial transcriptomics localized NRF2-imprinted SPP1-positive macrophages around necrotic regions [96]. A breast-specific counterpart to that myeloid observation has now been reported: in TNBC, ACOD1 is enriched in macrophage-infiltrated tumors and confers ferroptosis resistance on tumor-associated macrophages through the KEAP1/NRF2 axis, promoting progression in co-culture and xenograft models with supporting evidence from the TCGA-BRCA and METABRIC cohorts [135]. Adipose tissue warrants particular emphasis in breast cancer, where the tumor is embedded within a fat pad rather than merely adjacent to one: the redox coupling reported between tumor and cancer-associated adipose tissue indicates that host antioxidant and NADPH-generating capacity forms part of the local metabolic environment rather than a passive background [133]. The direction of the stromal contribution is not uniform, however. Microenvironmental NRF2 activation can restrict rather than support the progression of NRF2-activated tumors [136], whereas NRF2 activation in cancer-associated fibroblasts [132] and in the myeloid compartment [96] favors tumor growth and blunts responses to checkpoint blockade. Compartment, therefore, determines the sign of the effect, and heterogeneity in NRF2 activity across stromal, adipose, and immune populations is likely an underappreciated variable in both biomarker interpretation and systemic pathway modulation. Together, these findings show that bulk NRF2 measurements can aggregate malignant, stromal, adipose, and immune signals with opposing biological consequences, reinforcing the need for compartment-resolved biomarkers and for caution with systemic pathway modulation.

## 7. Therapeutic Targeting of NRF2: Direct Inhibition, Indirect Vulnerabilities, and Chemoprevention Paradoxes

There is no single correct way to act on NRF2 in breast cancer (Figure 3). The same pathway that may be activated to protect normal tissue and prevent transformation may become a therapeutic vulnerability once a tumor has become NRF2-dependent.

### 7.1. When to Activate NRF2: Chemoprevention and Tissue Protection

NRF2 activation is a defensible strategy in two distinct settings: cancer prevention before the onset of malignancy and protection of normal tissues from treatment-related toxicity. The first is chemoprevention. At low, non-cytotoxic doses, NRF2 activators induce a program that is overwhelmingly NRF2-dependent—in vivo, most altered gene expression and the chemoprotective efficacy of enzyme inducers are lost in *Nrf2*-knockout animals—enhancing carcinogen detoxification and protecting genomic integrity in normal and preneoplastic cells [137]. Dietary isothiocyanates such as sulforaphane are prototypical agents; a randomized trial of a broccoli-sprout beverage demonstrated sustained detoxification of environmental carcinogens in humans, establishing pharmacodynamic target engagement [138]. The second setting is mitigation of treatment toxicity: because NRF2 protects normal tissue from oxidative injury, its transient activation may shield non-malignant organs from the toxicities of cytotoxic therapy. Importantly, this targeted, transient pharmacological activation of NRF2 for chemoprevention is mechanistically distinct from chronic, broad-spectrum dietary antioxidant supplementation, which lacks NRF2-pathway selectivity and, in some settings, has been associated with harm rather than benefit; the two should not be conflated.

The paradox that constrains both uses recapitulates the central theme of this review. The very activation that protects a normal cell can, in a tumor that is already NRF2-high, promote tumor survival and therapy resistance (Section 3 and Section 6); sulforaphane itself, chemoprotective before initiation, can promote the growth of already initiated tumors in vivo [139]. Two cautions follow. First, systemic NRF2 activators carry off-target safety liabilities, as illustrated by the cardiovascular signal that halted a phase 3 program of the potent activator bardoxolone methyl in a non-oncological indication [15]. Second, and more subtly, the assumption that NRF2 activation is uniformly adverse in breast cancer is itself too simple: transcriptomic analyses across large cohorts (METABRIC and others) found that high *NFE2L2* expression was associated with better survival, lower grade, and an inflamed, immune-infiltrated phenotype in ER-positive/HER2-negative disease [68].

### 7.2. When to Inhibit NRF2: The Candidate Redox-Addicted Advanced Tumor

NRF2 inhibition is rational in the mirror-image setting: an established, NRF2-high tumor in which the pathway has become a survival and resistance program. Pharmacological proof of concept for NRF2-associated signaling in breast cancer has been reported: in HER2-positive breast cancer cells, brusatol suppressed growth by repressing the NRF2/HO-1 and HER2–AKT/ERK1/2 axes and synergized with trastuzumab, with the combination reducing tumor burden in xenografts while trastuzumab in turn amplified brusatol-induced ROS accumulation and apoptosis [69]. The subset remains proposed rather than demonstrated. Table 3 summarizes the NRF2-directed agents and modulator classes relevant to breast cancer, together with their molecular targets, the level of breast cancer-specific evidence, their principal limitations, and their clinical maturity.

### 7.3. How to Target NRF2 Safely: Three Routes and Their Maturity

#### 7.3.1. Direct Inhibition: Promising but Pharmacologically Immature

Direct NRF2 inhibitors remain preclinical tools rather than clinic-ready drugs, as recent surveys of NRF2 activators and inhibitors emphasize [9,147,148], and the two most-cited compounds illustrate why. Brusatol, the first and most potent reported NRF2 inhibitor, reduces NRF2 within 2–4 h in vitro and sensitizes a broad range of cancers to chemotherapy [149]; mass-spectrometry profiling subsequently showed, however, that it suppresses global protein synthesis and lowers NRF2 in all cell lines regardless of *KEAP1*/*NFE2L2* status—behavior consistent with a general translation inhibitor rather than a specific NRF2 antagonist [140]. ML385 is a more selective tool: it binds the Neh1 DNA-binding/sMAF-interaction region of NRF2, blocks target-gene transcription, and, in KEAP1-mutant NSCLC, potentiates carboplatin in orthotopic models [141]. However, its most extensively characterized activity is in KEAP1-deficient preclinical models; it remains a probe compound, not an approved therapeutic, and its evidence in breast cancer is limited to the cell-level re-sensitization experiments cited earlier. Overall, direct NRF2 inhibition has not yet yielded a clinically usable, breast cancer-validated drug—a gap that remains a major barrier to therapeutic translation.

#### 7.3.2. Indirect Targeting of NRF2-Conditioned Dependencies

A more tractable strategy exploits the dependencies that NRF2 activation creates rather than inhibiting the transcription factor itself. Three such approaches are relevant to breast cancer.

First, upstream kinase targeting may reduce the signaling that sustains NRF2 activity. In TNBC, receptor and non-receptor tyrosine kinases interact with the NRF2 pathway, and the interplay among MET, SRC, and NRF2 was shown to be therapeutically targetable—pharmacological inhibition of MET, SRC, or NRF2 enhanced paclitaxel sensitivity in TNBC cell lines and, importantly, in patient-derived organoids lacking detectable *SRC*, *NFE2L2*, or *KEAP1* mutations [142]. These findings provide proof of principle that approved tyrosine-kinase inhibitors can be repurposed as indirect modulators of the NRF2 pathway, although the evidence remains preclinical and requires validation in molecularly stratified breast cancer models and clinical cohorts.

Second, downstream ferroptosis-defense targeting may exploit the reliance of NRF2-active tumors on lipid peroxide control. NRF2 sustains the SLC7A11–glutathione–GPX4 axis by driving cystine uptake, glutathione synthesis, and the detoxification of phospholipid hydroperoxides; SLC7A11, GPX4, and glutathione synthesis therefore represent potential therapeutic nodes through which ferroptotic sensitivity could be restored in NRF2-high tumors [115,116,150]. Inhibiting these defenses may allow lipid peroxides to accumulate, re-exposing resistant cells to ferroptosis-inducing chemotherapy or radiotherapy. Because some NRF2-addicted TNBC cells also rely on glutathione-independent protective outputs, however, blocking the SLC7A11–glutathione–GPX4 axis alone may be insufficient—an argument for biomarker-guided combinations rather than uniform downstream inhibition across TNBCs.

Third, NQO1-directed bioactivation may convert an NRF2-regulated detoxification enzyme into a tumor-selective vulnerability. In *KEAP1*-deficient tumors, high NQO1 expression was identified as a pharmacologically exploitable dependency linked to ferroptosis induction and an anti-tumor immune response, with NQO1 protein proposed as a candidate predictive biomarker of immunotherapy sensitivity [145]. Consistent with this, the NQO1-bioactivatable prodrug β-lapachone undergoes an NQO1-dependent futile redox cycle in NQO1-high cells, generating tumor-selective ROS, exhausting NAD^+^ and ATP, and engaging a base-excision-repair catastrophe [151,152]; it also drives immunogenic cell death and HMGB1 release and, when combined with anti-PD-L1, reactivates CD8^+^ T-cell responses to overcome checkpoint-blockade resistance [146]. Much of this mechanistic and immunotherapy evidence derives from *KEAP1*-deficient non-breast cancer models; extension to NRF2-high breast cancer requires breast cancer-specific validation.

Collectively, these approaches target the signaling inputs, ferroptosis-defense outputs, and enzymatic liabilities of NRF2 activation.

#### 7.3.3. Combination and Biomarker-Guided Selection

The therapeutic potential of NRF2-directed strategies, therefore, depends jointly on the choice of combination and on the selection of tumors. With respect to combination, preclinical studies support NRF2-directed or dependency-directed targeting principally as a sensitizing strategy rather than as monotherapy, in combination with cisplatin [72], carboplatin [141], paclitaxel [70,142], radiotherapy [71], HER2-targeted trastuzumab [69], and checkpoint blockade [145,146], although the breast cancer-specific evidence varies across these combinations. With respect to selection, every strategy above presumes the ability to identify NRF2-dependent disease, which returns the argument to the biomarker problem—nuclear NRF2 protein, reduced cytoplasmic KEAP1 protein, a target-gene signature, and candidate markers such as NQO1 [145] rather than the *NFE2L2* transcript alone. Table 4 separates breast cancer-specific evidence from extrapolated evidence for the therapeutic strategies discussed here and in Section 8.

## 8. Translational Framework: Patient Selection, Combination Therapy, and Testable Predictions

The biology and pharmacology reviewed above support a translational framework in which NRF2-directed therapy in breast cancer is most plausibly developed as a stratified, combination-based intervention, organized around three linked problems—who to treat, what to combine, and how to identify the appropriate patients.

### 8.1. Patient Stratification: Prioritizing the Candidate Redox-Addicted Subset

The recurring claim of this review is that the target population is not all “TNBC,” but a candidate redox-addicted subset within it (Section 4.4), and the practical task is to identify those tumors prospectively. Three convergent features nominate candidates. First, protein-level evidence of NRF2 activation: a poor-prognosis protein phenotype characterized by high nuclear NRF2 and low cytoplasmic KEAP1 is identifiable by immunohistochemistry and has already been shown to stratify survival in operable TNBC [65]. Second, a mesenchymal/lipid-metabolic phenotype: high SLC7A11/xCT-driven cystine dependence in the mesenchymal stem-like state [66], together with preferential ACSL4 expression in basal-like breast cancer cell lines, predicts ferroptosis sensitivity [67]. Third, functional evidence of active NRF2 signaling and ferroptosis-related vulnerability: NQO1 protein has emerged as a candidate biomarker in NRF2-active, KEAP1-deficient tumors, although this evidence is largely derived from non-breast cancer models [145]. A triple-negative tumor negative across them should not automatically be regarded as NRF2-dependent. Here too, the subset is proposed, not demonstrated.

### 8.2. Combination Strategies

Three combination strategies emerge from the preclinical breast cancer evidence, which supports NRF2-directed agents as sensitizers rather than as stand-alone treatments. The first pairs NRF2 or dependency inhibition with the modality whose resistance it reverses—cisplatin [72], carboplatin [141], paclitaxel [70,142], HER2-targeted trastuzumab [69], or radiotherapy [71]—thereby restoring oxidative or ferroptotic killing. The second is ferroptosis induction combined with immune-checkpoint blockade in the candidate redox-addicted subset: because NQO1-driven ferroptosis can trigger immunogenic cell death and reactivate T-cell responses, NQO1 bioactivation (e.g., β-lapachone) plus anti-PD-(L)1 is a mechanistically grounded pairing [145,146], with the unresolved caveat that NRF2 also operates in normal immune cells and systemic inhibition risks immune toxicity (Section 6.5). The third targets the metabolic dependency of NRF2-activated cells: genomic KEAP1 loss imposes a dependence on glutaminolysis in lung adenocarcinoma, rendering such tumors sensitive to glutaminase inhibition [143], and the glutaminase inhibitor telaglenastat (CB-839) advances into biomarker-selected clinical trials in *KEAP1/NFE2L2*-mutant disease, combined with pembrolizumab and chemotherapy in NSCLC (KEAPSAKE; NCT04265534) [144]—though this trial was terminated early for lack of clinical efficacy [144], a cautionary result that tempers expectations for the metabolic-dependency strategy.

### 8.3. The Biomarker Problem and a Conceptual NRF2-Activation Score

Every element above depends on reliably measuring NRF2 activity, and this is difficult for four reasons: the *NFE2L2* transcript is a poor surrogate, antibody performance varies, bulk profiling conflates tumor and stroma, and activity is spatially structured within a tumor, so a single-region sample may not represent it. The clinical trial experience reinforces the difficulty: across NRF2-targeting agents, no single biomarker has proven sufficient to define pharmacodynamic action, and composite panels spanning target-gene expression, oxidative stress, and metabolomic readouts have been required [15]. The discordance—high *NFE2L2* transcript tracking better outcome in ER-positive disease [68] while high nuclear NRF2 protein tracks worse outcome in TNBC [65]—is itself a warning against any single-marker definition.

Against this background, we offer, explicitly as a hypothesis-generating conceptual framework rather than a validated assay, a composite NRF2-activation score (Box 1) that would integrate (i) nuclear NRF2 protein, (ii) reduced cytoplasmic KEAP1 protein expression, (iii) an NRF2 target-gene signature, and (iv) ferroptosis-defense markers. A therapy-resistant phenotype is deliberately excluded from the score because it is the outcome the score is intended to predict; it is recorded separately as a validation-context variable. Signatures of this type already carry prognostic information in other NRF2-driven cancers, though none have been validated in breast cancer [153]. One internal-consistency point deserves emphasis. The shared appearance of *SLC7A11* and *NQO1* across the target-gene signature (iii) and ferroptosis-related readout (iv) is intentional: they capture overlapping but distinct dimensions of pathway activity and ferroptotic vulnerability, not double-counting.

We stress that such a score is, at present, conceptual: it requires construction and prospective validation against clinical endpoints in defined breast cancer cohorts, using analyses able to distinguish malignant-cell NRF2 activity from microenvironmental NRF2 activity (Section 4.5)—a distinction with functional significance, since microenvironmental NRF2 activation can itself restrict the progression of NRF2-activated tumors [136]. Two practical constraints bound any such effort. First, to our knowledge, no regulatory agency has approved a companion diagnostic for nuclear NRF2, and no scoring threshold, antibody clone, or staining protocol has been prospectively validated against a clinical endpoint in breast cancer; the immunohistochemical readouts on which the score depends are therefore research-grade assays, not clinical ones. Second, adding immunohistochemistry, target-gene profiling, or spatial analysis for the sole purpose of treatment assignment carries a real burden in turnaround time, cost, and tissue, and a stratification strategy that cannot be executed within routine diagnostic workflows will not reach patients, however well it performs analytically. Both constraints argue for developing the simplest combination of markers that retains predictive value, rather than the most complete one.

Box 1 **Proposed composite NRF2-activation score (conceptual; hypothesis-generating).**

A proposed treatment-selection framework for “NRF2-high breast cancer” that is robust to the limitations of any single biomarker. It integrates:

(i) (Nuclear NRF2 protein—immunohistochemistry.
(ii) Reduced cytoplasmic KEAP1 protein expression—immunohistochemistry.
(iii) NRF2 target-gene signature—e.g., *NQO1*, *GCLC*, *GCLM*, *SLC7A11*, *TXNRD1*.
(iv) Ferroptosis-defense markers—e.g., SLC7A11, GPX4, NQO1.

Not a component: a therapy-resistant phenotype is deliberately excluded because it is the outcome the score is intended to predict; it is recorded separately as a validation-context variable.

Intended use: prospective identification of the candidate redox-addicted TNBC subset for NRF2-directed or ferroptosis-inducing therapy.

Validation status: requires construction and prospective validation against clinical endpoints, ideally with single-cell or spatially resolved analyses to distinguish malignant cells from microenvironmental NRF2 activity before any clinical application (Section 8.3).

Current status: conceptual framework only; not a validated clinical assay.

*A biomarker-development roadmap.* Operationalizing this composite phenotype requires a staged validation program. (i) Analytical validation: antibody specificity, reproducible nuclear-versus-cytoplasmic scoring, control of batch effects, and prespecified positivity cutoffs. (ii) Biological validation: correlation of nuclear NRF2 immunohistochemistry with NRF2 target-gene signatures, lipid-peroxidation markers, cystine dependence, and experimental ferroptosis sensitivity. (iii) Clinical validation: association with treatment-specific outcomes in retrospective, therapy-annotated cohorts rather than heterogeneous patient populations. (iv) Predictive validation: prospective biomarker-stratified clinical trials or pharmacodynamic testing in patient-derived organoid and xenograft models to demonstrate that biomarker-positive tumors preferentially respond to NRF2- or ferroptosis-directed intervention. (v) Compartment resolution: distinction of malignant-cell NRF2 activity from stromal and immune NRF2 activity using spatial transcriptomics or multiplex immunohistochemistry, because bulk profiling conflates these signals.

### 8.4. Open Questions and Testable Predictions

The proposed framework generates a series of testable predictions that distinguish it from a generic view that “NRF2 matters in cancer” and are, in principle, experimentally falsifiable:

P1 (stratification). Among TNBC, responses to NRF2-pathway or ferroptosis-directed therapy will be concentrated in tumors scoring high on the composite of Section 8.3 and will be poorly predicted by triple-negative status alone.

P2 (transition point). Within an individual ER-positive tumor, NRF2 activity will rise upon and as a consequence of tamoxifen exposure [64]. Whether other endocrine agents produce the same induction is untested. Pre-treatment NRF2 status will predict this acquired activation less well than on-treatment sampling.

P3 (cross-resistance). Tumors that acquire NRF2-high status under one oxidative-stress-inducing therapy will display cross-resistance to others (endocrine → radiation [64]; ferroptosis evasion → chemotherapy/radiotherapy [70,71]), and NRF2 inhibition will reverse this cross-resistance.

P4 (combination specificity). Ferroptosis-inducing or NQO1-bioactivating agents combined with checkpoint blockade will specifically benefit the candidate redox-addicted subset, but the therapeutic window may be constrained by the effects of systemic NRF2 inhibition on normal immune-cell function (Section 6.5).

P5 (chemoprevention boundary). NRF2 activators will protect normal and preneoplastic breast tissue but will provide no benefit—and may be harmful—in tumors already scoring NRF2-high, predicting that the benefit of chemopreventive activation is confined to the premalignant window and that its evaluation must be stage-specific.

These predictions also highlight the key unanswered questions: what threshold of the composite score discriminates responders; whether the transition point can be detected non-invasively.

### 8.5. Limitations of This Review

Several limitations of this review should be acknowledged. First, this is a narrative rather than a systematic review, so despite explicit appraisal criteria, the selection of studies remains susceptible to selection and publication bias. Second, several central claims for the candidate redox-addicted TNBC subset rest on a limited clinical anchor—most notably a single operable TNBC tissue microarray cohort—alongside cell-line and mechanistic data, and therefore support an association and a biologically plausible mechanism rather than a prospectively validated dependency. Third, the discussion of immunotherapy—and, as flagged in Section 6, several other mechanistic elements of the framework—is largely extrapolated from non-breast (predominantly lung and hepatocellular) cancers and should be read as hypothesis-generating. Fourth, the proposed composite NRF2-activation score is conceptual and has not been analytically or clinically validated. Finally, the TNBC treatment landscape is evolving rapidly, so specific regimens cited here may be superseded; the principal contribution is the biological and evidentiary framework rather than any particular drug recommendation.

## 9. Conclusions

The duality of NRF2 in breast cancer is real, but it is not a property of “breast cancer” as a whole. It is conditioned by molecular subtype, disease stage, and the specific therapy applied. Before transformation, NRF2 activation is chemopreventive. In established disease, persistent activation becomes a tumor-fitness and resistance program, but the evidence for such dependence varies substantially across subtypes: limited and largely therapy-associated in luminal disease, under-characterized but mechanistically plausible in HER2-enriched disease, and most strongly supported in a putative redox-addicted subset of TNBC.

Within that subset, NRF2-driven suppression of ferroptosis represents a major, therapeutically tractable convergence point—among several NRF2-mediated resistance outputs—linking the candidate redox-addicted state to resistance across endocrine therapy, chemotherapy, radiotherapy, and, plausibly, immunotherapy, even though NRF2 also contributes to resistance through parallel outputs such as ROS buffering, glutathione synthesis, xenobiotic detoxification, drug-efflux induction, DNA-repair support, and metabolic reprogramming. The therapeutic implication is correspondingly conditional and, we stress, hypothesis-generating: NRF2 activation is rational only to protect normal tissue and prevent transformation, whereas inhibition—or, more tractably, circumvention through its upstream kinases and downstream ferroptotic dependencies—warrants investigation primarily in tumors with demonstrable functional NRF2 dependence.

Crucially, the proposed redox-addicted TNBC subset and composite NRF2-activation score remain hypotheses for prospective validation, not currently validated clinical entities. Their value will depend on demonstrating co-occurrence of the proposed protein, metabolic, lipid, and ferroptosis-defense features within individual tumors and showing that these features predict treatment response beyond standard clinicopathologic and molecular variables. If validated prospectively, this framework could provide a practical foundation for integrating NRF2-directed therapy into precision medicine for breast cancer.

## Figures and Tables

**Figure 1:**
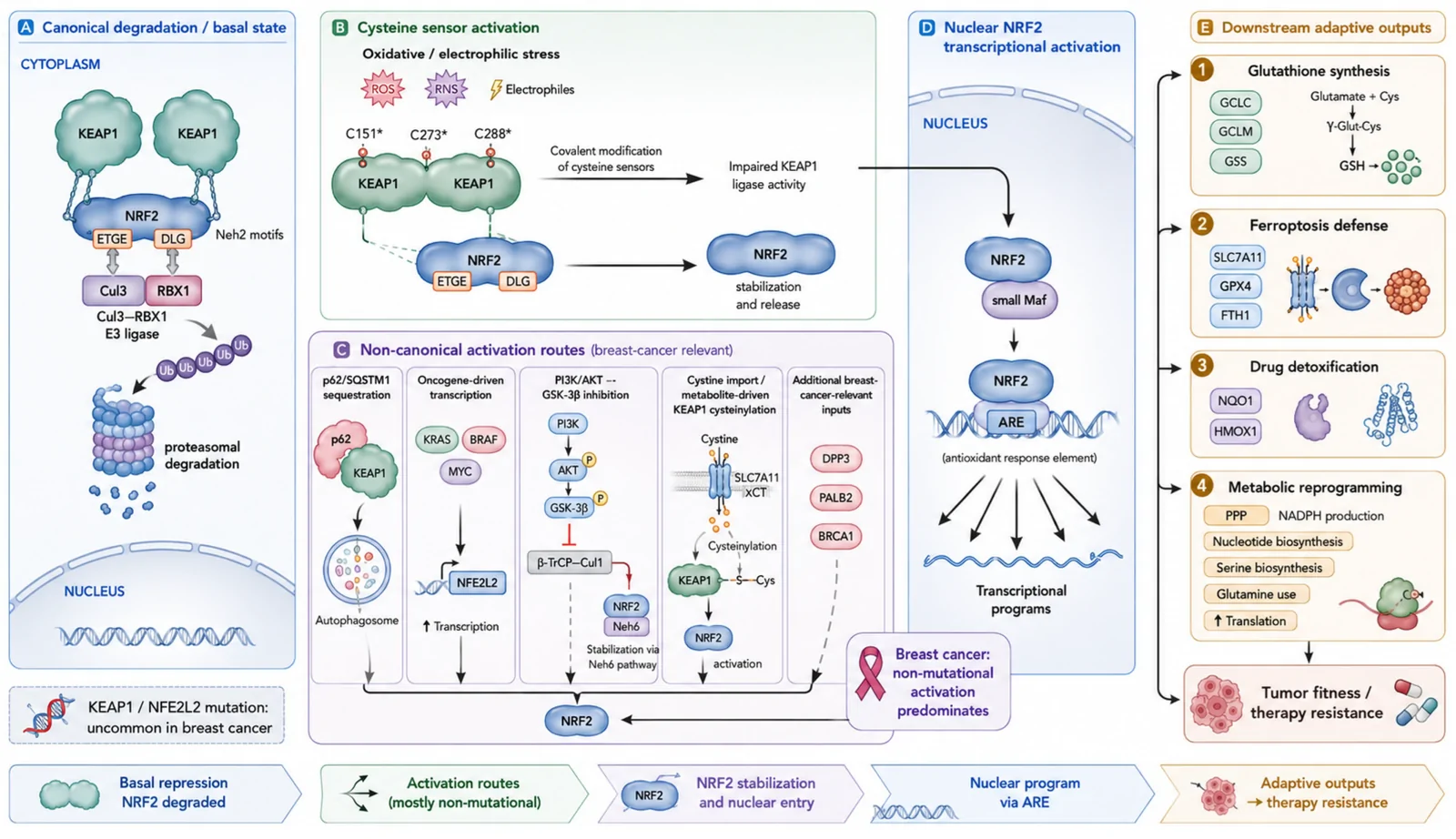
Architecture of the NRF2–KEAP1 axis and its predominantly non-mutational activation in breast cancer. (**A**) Canonical degradation. A KEAP1 homodimer engages NRF2 through the high-affinity ETGE and low-affinity DLG motifs of the Neh2 domain and presents it to the Cul3–RBX1 E3 ubiquitin ligase for polyubiquitination (Ub) and proteasomal degradation. (**B**) Cysteine-sensor activation. ROS, reactive nitrogen species (RNS), and electrophiles modify the sensor cysteines of KEAP1 (asterisks; Cys151, Cys273, Cys288), impairing ligase activity so that newly synthesized NRF2 escapes degradation and enters the nucleus. (**C**) Non-canonical activation routes (breast cancer-relevant). In breast cancer, NRF2 is activated predominantly through non-mutational routes that converge on NRF2 stabilization: (i) p62/SQSTM1-mediated sequestration of KEAP1 into autophagosomes; (ii) oncogene-driven upregulation of *NFE2L2* (KRAS, BRAF, MYC); (iii) PI3K/AKT-mediated inhibition of GSK-3β, disabling the β-TrCP–Cul1 degron (Neh6 pathway); (iv) cystine import via SLC7A11 (xCT) with metabolite-driven cysteinylation of KEAP1 (KEAP1–S–Cys); and (v) additional breast cancer-relevant inputs (DPP3, PALB2, BRCA1). Somatic *KEAP1/NFE2L2* mutations—the dominant activating mechanism in lung and several other cancers—are uncommon in breast cancer (lower left). (**D**) Nuclear transcriptional activation. Stabilized NRF2 heterodimerizes with small Maf proteins and binds antioxidant response elements (AREs) in target-gene promoters. (**E**) Downstream adaptive outputs. NRF2-driven programs include (1) glutathione synthesis (GCLC, GCLM, GSS; Glu + Cys → γ-Glu-Cys → GSH); (2) ferroptosis defense (SLC7A11, GPX4, FTH1); (3) drug detoxification (NQO1, HMOX1); and (4) metabolic reprogramming (pentose phosphate pathway/NADPH production, nucleotide and serine biosynthesis, glutamine utilization, and enhanced translation). Solid arrows denote established steps and dashed arrows denote inferred or context-dependent steps; asterisks mark the reactive sensor cysteines of *KEAP1*. ARE, antioxidant response element; BRCA1, breast cancer type 1 susceptibility protein; Cul3, Cullin 3; DPP3, dipeptidyl peptidase 3; FTH1, ferritin heavy chain 1; GCLC/GCLM, glutamate–cysteine ligase catalytic/modifier subunit; GPX4, glutathione peroxidase 4; GSH, glutathione; GSK-3β, glycogen synthase kinase 3β; GSS, glutathione synthetase; HMOX1, heme oxygenase 1; KEAP1, Kelch-like ECH-associated protein 1; *NFE2L2*/NRF2, nuclear factor erythroid 2-related factor 2; NQO1, NAD(P)H:quinone oxidoreductase 1; PALB2, partner and localizer of BRCA2; PPP, pentose phosphate pathway; RBX1, RING-box protein 1; RNS, reactive nitrogen species; ROS, reactive oxygen species; SLC7A11 (xCT), cystine/glutamate antiporter; Ub, ubiquitin.

**Figure 2:**
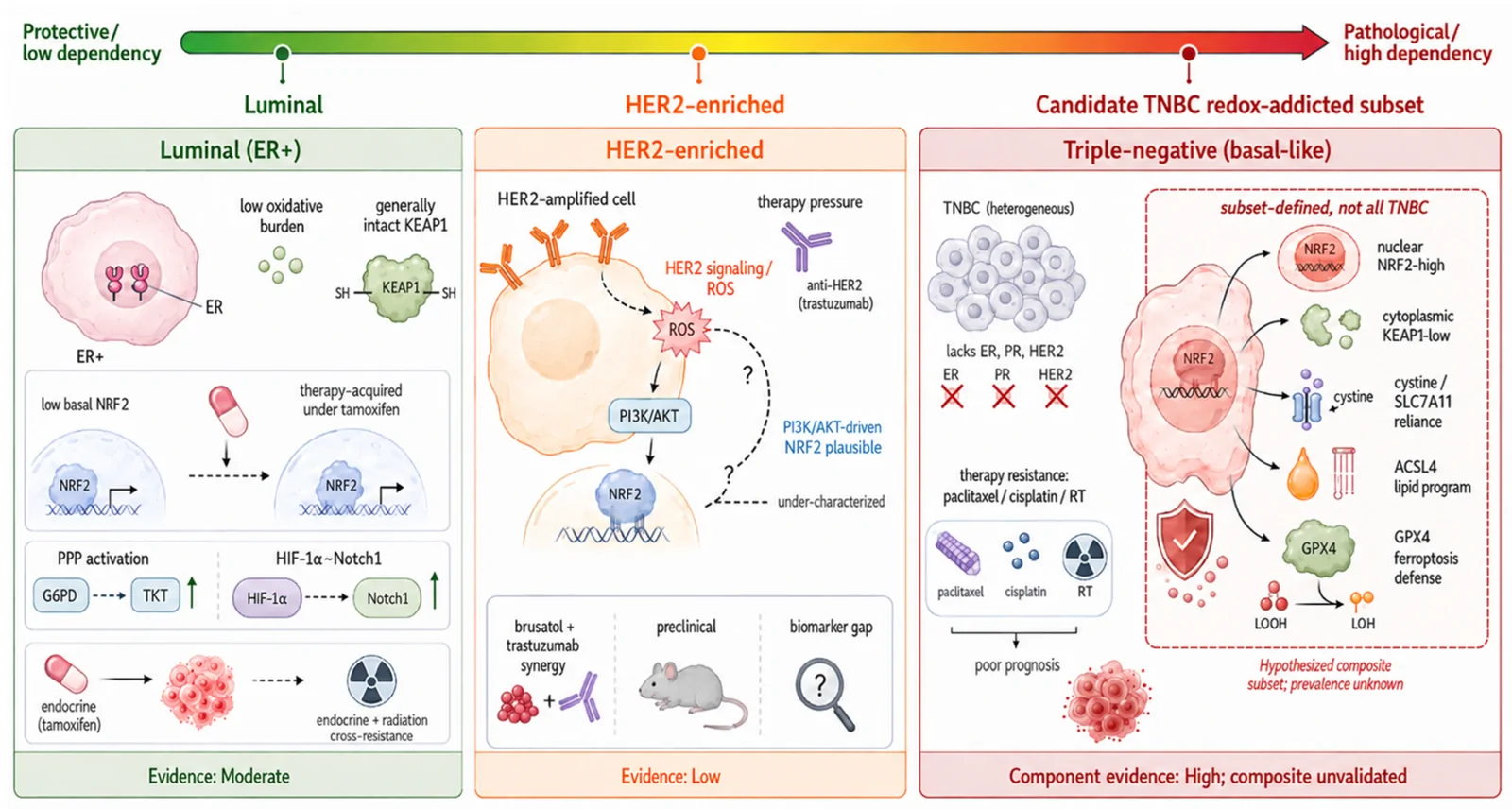
Subtype-conditioned model of NRF2 biology in breast cancer. The protective-to-oncogenic behavior of NRF2 is depicted as a continuum (top bar) rather than as a categorical property: dependency is low and largely therapy-acquired in luminal disease, under-characterized in HER2-enriched disease (evidence gap), and strongest within a candidate redox-addicted subset of triple-negative breast cancer (TNBC) (nuclear NRF2-high with cytoplasmic KEAP1-low, cystine–SLC7A11 reliance, basal-like ACSL4 lipid program, and active GPX4-dependent ferroptosis defense). Each panel summarizes the biological context, therapeutic pressure, and current level of evidence for each subtype. Evidence note: the model organizes existing evidence and does not imply that molecular subtype alone determines NRF2 dependency; grades reflect converging prognostic and mechanistic data rather than prospective clinical validation. Co-occurrence of these features within individual tumors has not been demonstrated, and the fraction of TNBC involved is unknown. Arrows denote the direction of the indicated process; a question mark marks a step that is un-der-characterized in that subtype.

**Figure 3:**
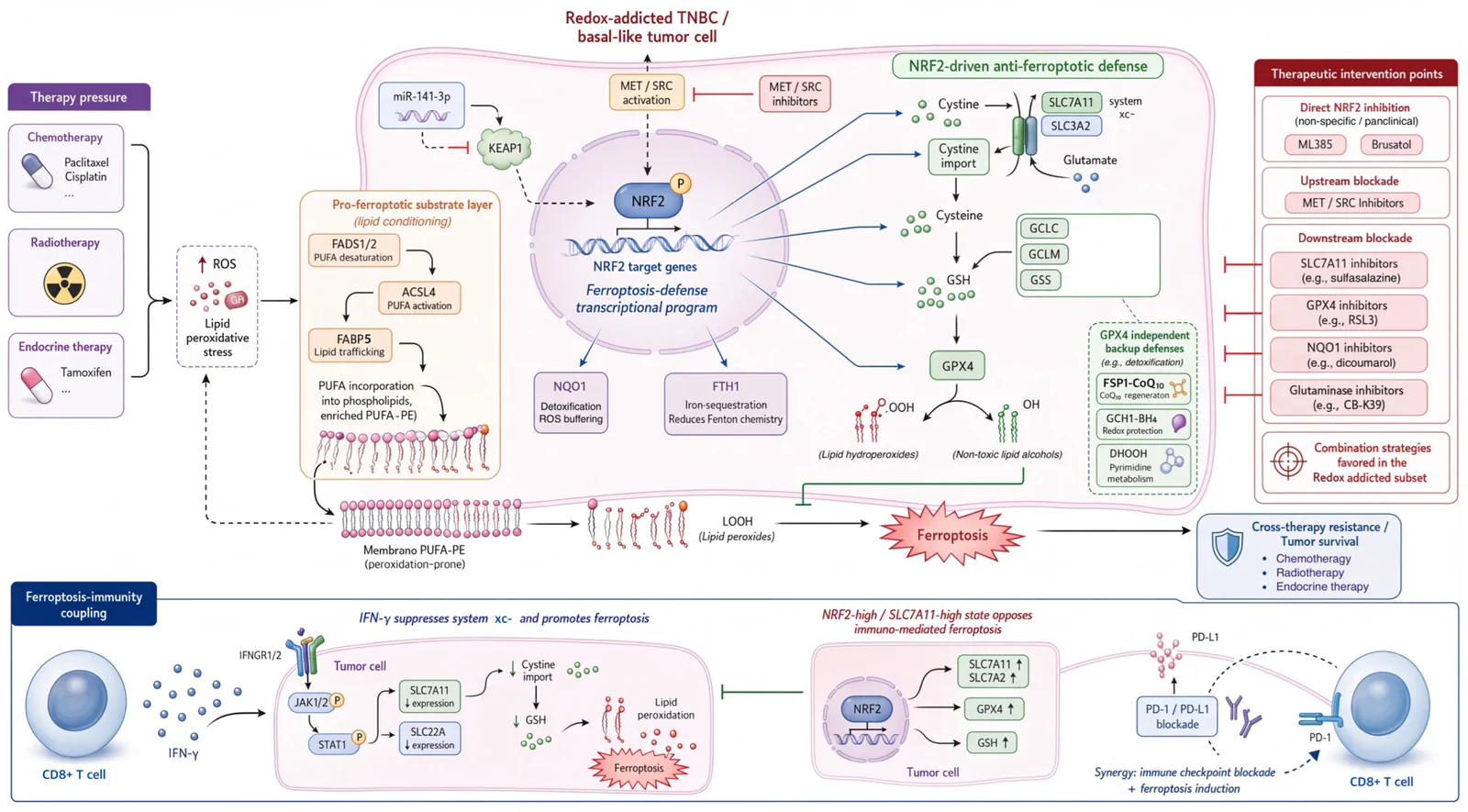
NRF2-driven ferroptosis-resistance circuit and intervention points. NRF2 transcriptionally sustains a ferroptosis-defense network centered on the SLC7A11–glutathione–GPX4 axis (with contributions from NQO1 and FTH1), thereby suppressing lipid peroxidation and ferroptosis. This network counteracts the pro-ferroptotic lipid-substrate layer (ACSL4/PUFA-PE, FADS1/2, FABP5) and buffers the oxidative lethality of chemotherapy, radiotherapy, and endocrine therapy, thereby promoting cross-therapy resistance. Pharmacological intervention points act directly on NRF2 (ML385, brusatol; non-specific/preclinical), upstream (MET/SRC), or downstream (SLC7A11, GPX4, NQO1, glutaminase), most effectively in combination strategies and within the candidate redox-addicted subset. Ferroptosis is additionally coupled to anti-tumor immunity (lower panel). Evidence note: the SLC7A11–GPX4 ferroptosis-defense core and its role in chemotherapy and radiotherapy resistance rest on breast cancer data, whereas the immunotherapy component represents a mechanistically plausible extrapolation, derived chiefly from non-breast tumors, that requires breast cancer-specific validation. Arrows denote the direction of the indicated process; blunt-ended connectors denote inhibition.

**Table 1:** Comparison of the present review with major prior reviews of NRF2 biology and ferroptosis in cancer.

Review	Primary Scope (Ferroptosis Emphasis)	Breast Cancer Subtype Resolution	Therapy-Resistance Framework	Biomarker/Patient-Selection Framework	Relevant Gap Addressed for the Present Review
Sporn & Liby, 2012 [12]	NRF2 duality in cancer; “importance of context” (pre-ferroptosis)	None (pan-cancer)	General	None	No breast-subtype or ferroptosis resolution
Menegon et al., 2016 [10]	Dual protective/oncogenic functions of NRF2 (minimal ferroptosis)	None (pan-cancer)	General resistance	None	No subtype-conditioned or ferroptosis framework
Rojo de la Vega et al., 2018 [11]	NRF2 and the hallmarks of cancer (emerging ferroptosis)	None (pan-cancer)	Broad, mechanism-level	Conceptual	Not breast-specific; no redox-addicted-subset definition
Cuadrado et al., 2019 [9]	Therapeutic targeting of NRF2/KEAP1 (limited ferroptosis)	None (pan-cancer/disease)	Drug-resistance-oriented	Druggability focus	Not breast-focused; no patient-selection logic
Anandhan et al., 2020 [14]	NRF2–ferroptosis-defense axis (central ferroptosis)	None (pan-cancer)	Ferroptosis-centered	Mechanistic	Not breast-subtype-resolved; no clinical-stratification framework
Yagishita et al., 2020 [15]	Landscape of NRF2 biomarkers in clinical trials (limited ferroptosis)	None (pan-cancer)	Trial-oriented	Biomarker landscape	Not breast-specific; not linked to a ferroptosis-defined subset
Present review	NRF2–KEAP1 as a subtype-conditioned redox vulnerability in breast cancer (central, integrated ferroptosis)	TNBC subset-resolved (nuclear NRF2 + KEAP1 loss)	Graded by treatment modality; breast-specific vs. extrapolated separated	Composite NRF2-activation score + staged development roadmap	Proposes and grades a candidate protein-defined redox-addicted TNBC subset (nuclear NRF2 + KEAP1 loss) with therapy-resistance and biomarker logic; unlike recent breast/TNBC ferroptosis reviews (Mokhtarpour et al., 2024 [13]), it separates breast-native from extrapolated evidence and adds a composite biomarker score

Grade definitions and appraisal criteria are given in “Evidence grading”.

**Table 2:** Evidence scorecard for NRF2/KEAP1 status, NRF2-high phenotype, and functional dependency across breast cancer subtypes.

Feature	Luminal (ER^+^)	HER2-Enriched	TNBC (Basal-Like)
*NFE2L2/KEAP1* somatic mutation	Rare, <10% (H) [6] *[1 breast genomic cohort (TCGA)]*	Rare; highest overall TMB but not *NFE2L2*/*KEAP1*-driven (H) [6] *[1 breast genomic cohort (TCGA)]*	Rare; activation largely non-mutational (H) [6] *[1 breast genomic cohort (TCGA)]*
NRF2 protein/activity	Low basal; therapy-acquired via tamoxifen (H) [64] *[1 preclinical + cell]*	Under-characterized; PI3K/AKT-driven plausible; NRF2 protein now measured in HER2^+^ tumors (M) [6] *[1 retrospective paired-biopsy cohort, n = 75]*	High in a subset; nuclear NRF2 accumulation (H) [65] *[1 retrospective TMA, n = 203]*
KEAP1 status	Generally intact (M) *[0 direct; inferred]*	Under-characterized (L) *[0 direct; inferred]*	Reduced cytoplasmic KEAP1 protein in poor-prognosis subset (H) [65]; cystine-cysteinylation in MSL (H) [66] *[1 retrospective TMA, n = 203 + 1 preclinical study]*
NRF2 target-gene signature	PPP axis inducible: G6PD/HIF-1α/Notch1 (M) [57] *[1 preclinical]*	Not subtype-resolved (L) *[0 direct]*	OSGIN1/SRXN1/AKR1B10 in MSL (H) [66]; glycolytic (M) [57] *[2 preclinical]*
Cell-line functional evidence	MCF-7 NRF2–PPP–EMT (M) [57] *[1 cell]*	Sparse (L) *[0 direct]*	MSL cystine–NRF2 survival (H) [66]; basal-like ACSL4 (H) [67] *[2 cell]*
Patient survival association	Higher *NFE2L2* mRNA → better survival, immune-inflamed (M) [68] *[1 multi-cohort transcriptomic study]*	Not established (L)* [0 direct]*	Nuclear NRF2-high + KEAP1-low → worst OS/DFS, *n* = 203 (H) [65] *[1 retrospective TMA, n = 203]*
Therapy-resistance link	Endocrine → radiation cross-resistance (H) [64] *[1 preclinical]*	Anti-HER2 (brusatol + trastuzumab, preclinical); NRF2 upregulation associated with failure of pCR (M) [69] *[1 preclinical xenograft + 1 retrospective cohort, n = 75]*	Paclitaxel/cisplatin/RT; cancer stem cells (H) [70,71,72] *[3 preclinical + 1 retrospective cohort, n = 56]*
Ferroptosis relevance	Limited (M) *[0 direct; inferred]*	Not established (L)* [0 direct]*	High vulnerability in subset: ACSL4, xC−–GPX4 (H) [67,73] *[2 preclinical]*
Research priority/biological rationale (redox)	Low priority	Defined but under-powered—confirmation priority	Highest priority—candidate redox-addicted subset
Clinical readiness (Not validated/Exploratory/Candidate)	Not validated	Not validated	Not validated
Overall evidence strength (High/Moderate/Low)	Moderate (mechanistic, therapy-acquired)	Low to moderate (first direct human evidence; single retrospective cohort)	High for the component evidence; composite unvalidated

Grades (in parentheses) describe the directness and strength of the evidence available for each dimension. H (High) = the dimension is supported by direct breast cancer evidence of the type appropriate to that dimension, from a study designed to address it (a well-powered human cohort for prevalence or survival; functional manipulation in breast cancer models for mechanism), without contradictory evidence; M (Moderate) = supported by indirect, single-model, or partially concordant evidence, or by substantial extrapolation; L (Low) = sparse, absent, or wholly extrapolated evidence. The convergence criterion (human cohort plus protein or pathway evidence plus functional validation) applies only to the overall evidence strength row, not to the individual dimensions. Bracketed numbers are references. Bracketed italic tags give the number and type of key supporting studies for each entry; [0 direct] indicates that the entry rests on inference from other subtypes or tumor types rather than on breast cancer-specific data. Grades reflect the consistency and level of the available evidence, not the number of studies, so a High grade supported by a single cohort denotes internally consistent but not independently replicated evidence. A High grade denotes evidence appropriate to the dimension being graded and does not by itself establish a prospectively validated functional dependency in patients; clinical actionability remains Low across all subtypes. The two summary rows report these dimensions separately—overall evidence strength (High/Moderate/Low) and clinical readiness (Not validated/Exploratory/Candidate)—so that a High evidence grade is not read as clinical readiness. PPP, pentose phosphate pathway; MSL, mesenchymal stem-like; EMT, epithelial-to-mesenchymal transition; OS, overall survival; DFS, disease-free survival; TMB, tumor mutational burden; RT, radiotherapy.

**Table 3:** NRF2-pathway modulators relevant to breast cancer: mechanism, evidence, limitations, and clinical maturity.

Agent/Class	NRF2 Effect (Target)	Breast Cancer-Specific Evidence (Model)	Main Limitation	Clinical Maturity in Breast Cancer
Brusatol	Indirect ↓NRF2 protein (via global translation)	Tumor reduction + trastuzumab synergy in HER2^+^ xenograft [69]; enhances cisplatin in MCF-7 in vivo [72] (cell lines + xenograft)	Non-specific—global protein-synthesis inhibitor, not NRF2-selective [140]	Preclinical tool only (non-selective)
ML385	Direct—blocks NRF2 Neh1/sMAF DNA binding	Re-sensitizes TNBC stem cells to radiation [71]; reverses miR-141-3p paclitaxel resistance [70] (TNBC cells/CSC; NSCLC xenograft [141])	Probe compound; activity mainly in KEAP1-deficient context; no breast in vivo	Preclinical (tool compound)
MET/SRC inhibition (e.g., dasatinib)	Indirect ↓NRF2 via upstream kinase	+Paclitaxel sensitization in TNBC cells and patient-derived organoids [142]	Proof-of-principle; repurposing of approved TKIs	Preclinical (PDO); TKIs clinically available → repositioning candidate
Glutaminase inhibitor (telaglenastat/CB-839)	Indirect—targets NRF2 metabolic dependency (GLS)	Extrapolated from KEAP1-mutant lung adenocarcinoma [143]; biomarker-selected NSCLC trial (KEAPSAKE), terminated early [144]	Breast efficacy unproven; KEAPSAKE terminated for lack of efficacy	Other-cancer clinical evidence (not breast)
SLC7A11/xCT inhibitors (erastin, sulfasalazine)	Indirect—ferroptosis induction (system xc^−^)	TNBC/MSL xCT dependency and erastin sensitivity [66,73] (cell lines)	Ferroptosis sensitivity heterogeneous within TNBC; weak potency (sulfasalazine)	Preclinical (repurposed agents available)
GPX4 inhibitors (RSL3)	Indirect—ferroptosis induction (GPX4)	Basal-like/ACSL4-high TNBC subset sensitive [67,105] (cell lines)	No clinical GPX4 inhibitor; selectivity/PK unsolved	Preclinical (tool compound)
NQO1-bioactivatable (β-lapachone)	Exploits NRF2 output (NQO1) → tumor-selective ROS/ferroptosis	Cross-cancer ferroptosis + immunity [145]; β-lap + anti-PD-L1 [146] (non-breast xenograft)	Breast data lacking; NQO1-high dependence; hemolysis risk (class)	Other-cancer clinical; NQO1 a candidate biomarker
Sulforaphane (SFN)	Activator (KEAP1 Cys151 alkylation)	Human target engagement for chemoprevention [138]; enzyme-inducer chemoprevention is NRF2-dependent [137]	Paradox—may promote already initiated tumors [139]; benefit confined to premalignant window	Non-oncology/prevention clinical; biomarker-dependent
Bardoxolone methyl (CDDO-Me)	Potent activator (KEAP1 cysteine)	None breast-specific (cited for safety signal)	Cardiovascular safety signal (phase 3 halt, non-oncology) [15]	Cautionary—systemic-activator toxicity (non-breast)

Downward arrows denote a decrease in the indicated parameter. TKI, tyrosine-kinase inhibitor; PDO, patient-derived organoid; GLS, glutaminase; MSL, mesenchymal stem-like; PK, pharmacokinetics. Bracketed numbers are references. Clinical-maturity categories: breast cancer clinical/other-cancer clinical/non-oncology clinical/preclinical (breast)/tool compound only.

**Table 4:** Breast cancer-specific versus extrapolated evidence for NRF2-directed and NRF2-conditioned therapeutic strategies. Roman numerals in the final column give the evidence tier defined in Section 1.1: (i) direct breast cancer clinical evidence; (ii) breast cancer preclinical evidence; (iii) evidence obtained in other tumor types; (iv) a conceptual proposal of the present authors. No strategy listed here rests on tier (i), although tier (i) evidence now exists for the association between NRF2 protein and chemotherapy response (Section 4.3 and Section 6.2); the composite NRF2-activation score and predictions P1–P5 of Section 8 are tier (iv).

Strategy	Breast Cancer-Specific Evidence	Extrapolated/Non-Breast Evidence	Main Limitation	Current Interpretation
Brusatol + trastuzumab	HER2^+^ cells and xenografts [69]	Global translation inhibition/non-specific NRF2 suppression [140]	Brusatol is not NRF2-selective	(ii) Breast-specific preclinical proof of concept
NRF2 inhibition + cisplatin	Hypoxia-associated cisplatin sensitization in MCF-7/in vivo breast models [72]	Brusatol-mediated chemotherapy sensitization across cancer models [149]	Preclinical; inhibitor specificity	(ii) Breast-specific sensitization evidence
NRF2/MET/SRC targeting + paclitaxel	TNBC cell models and patient-derived organoids [70,142]	None required for core claim	No clinical validation; pathway context may vary	(ii) Breast-specific preclinical evidence
NRF2 inhibition + radiotherapy	TNBC and breast cancer stem-cell radiosensitization [71,99]	None required for core claim	Preclinical only	(ii) Breast-specific mechanistic evidence
Direct ML385 inhibition	Cell-level re-sensitization in breast models [70,71]	KEAP1-deficient NSCLC, including carboplatin sensitization [141]	Probe compound; not an approved therapy	(ii) and (iii) Not clinically ready
SLC7A11/GPX4 ferroptosis targeting	Basal-like/TNBC ferroptosis vulnerability and xCT dependency [67,73]	Broader ferroptosis-mechanism literature [115,116,150]	Heterogeneous sensitivity; glutathione-independent NRF2 outputs may persist	(ii) and (iii) Mechanistically supported; clinical validation absent
NQO1 bioactivation	None direct	KEAP1-deficient and pancreatic/non-breast models [145,146,151,152]	Breast-specific predictive biomarker evidence absent	(iii) Hypothesis-generating
Ferroptosis induction + checkpoint blockade	None direct	CD8^+^-ferroptosis and NQO1/checkpoint studies [129,145,146]	No direct breast combination data; host-compartment effects unresolved	(iii) Hypothesis-generating
Glutaminase inhibition in KEAP1/NRF2-high disease	None direct	KEAP1-driven NSCLC dependency and KEAPSAKE trial [143,144]	KEAPSAKE terminated early for lack of efficacy	(iii) Extrapolated; cautionary

## Data Availability

No new data were created or analyzed in this study. Data sharing is not applicable to this article. All studies synthesized here are published and publicly available, and the search strategy and eligibility criteria required to reproduce the literature identification are provided in Supplementary Note S2.

## Supplementary Materials

The following supporting information can be downloaded at: https://www.mdpi.com/article/10.3390/antiox15101223/s1, Supplementary Note S1, Intratumoral and spatial heterogeneity of NRF2 activity and its consequences for biomarker sampling; Supplementary Note S2, Literature search strategy, eligibility criteria, evidence-grading rubric, and claim-level evidence map; Table S1: Search sources, terms, combinations, and limits; Table S2: Eligibility criteria as opera-tionalized; Table S3: Evidence-grading rubric applied in Table 2 of the main text; Table S4: Claim-level evidence map for the principal statements of the review.

## Abbreviations

The following abbreviations are used in this manuscript:

ACSL4	acyl-CoA synthetase long-chain family member 4
ARE	antioxidant response element
BRCA1	breast cancer type 1 susceptibility protein
CSC	cancer stem cell
EMT	epithelial-to-mesenchymal transition
ER	estrogen receptor
FABP	fatty-acid-binding protein
GCLC	glutamate–cysteine ligase catalytic subunit
GCLM	glutamate–cysteine ligase modifier subunit
GPX4	glutathione peroxidase 4
GSH	glutathione
G6PD	glucose-6-phosphate dehydrogenase
HER2	human epidermal growth factor receptor 2
HMOX1	heme oxygenase 1
KEAP1	Kelch-like ECH-associated protein 1
MSL	mesenchymal stem-like
NFE2L2	nuclear factor erythroid 2-like 2
NQO1	NAD(P)H:quinone dehydrogenase 1
NRF2	nuclear factor erythroid 2-related factor 2
PD-L1	programmed death-ligand 1
PDO	patient-derived organoid
PUFA	polyunsaturated fatty acid
ROS	reactive oxygen species
SLC7A11	solute carrier family 7 member 11
TAM-R	tamoxifen-resistant
TKI	tyrosine-kinase inhibitor
TNBC	triple-negative breast cancer
xCT	cystine/glutamate antiporter light chain
