# Supplementary material for: Subtype-Conditioned NRF2–KEAP1 Signaling in Breast Cancer: A Narrative Review and Hypothesis-Generating Framework from Chemoprevention to Therapy Resistance
Source: Antioxidants (Basel). 2026 Sep 22;15(10):1223. doi: 10.3390/antiox15101223 (PMC13700803; doi:10.3390/antiox15101223)
Supplement: Supplementary file 1 [file antioxidants-15-01223-s001.zip › antioxidants-4484391-supplementary.pdf]

Supplementary Materials

Supplementary Note S1. Intratumoral and spatial heterogeneity of NRF2 activity and its consequences for biomarker sampling

Referenced from Section 4.5 of the main text. Reference numbers follow the main-text reference list.

A limitation that cuts across all subtype assignments in the main text is that NRF2 activity is not uniform within a tumor. The determinants invoked throughout this review — hypoxia, reactive oxygen species burden, nutrient and cystine availability, and therapy exposure — are themselves spatially structured. Each is most extreme in the poorly perfused tumor core and least so at the invasive margin. A tumor scored NRF2-high on a single block may therefore contain regions in which the pathway is quiescent, and a tumor scored NRF2-low may contain a focal NRF2-high population that could be missed by single-region sampling.

Direct evidence that the NRF2 activation state is spatially organized has recently become available. Spatial transcriptomic profiling across murine and human tumors resolves a gradient in which pro-inflammatory CXCL9-positive macrophages occupy the tumor periphery while NRF2-imprinted SPP1-positive macrophages accumulate around necrotic regions [96]. Within a single tumor, NRF2 activation state tracks distance from necrosis. Although that observation was made in the myeloid compartment, the stressors that drive it — hypoxia and oxidative burden — act on malignant cells in the same spatial pattern, and these shared spatial stressors provide a rationale to test whether malignant-cell NRF2 activity is similarly heterogeneous.

Two consequences follow for the composite score proposed in Section 8.3.

First, a single immunohistochemical score taken from a single region may misclassify a tumor in either direction. The reproducibility of any composite NRF2-activation score will therefore depend as much on sampling strategy as on antibody performance or scoring threshold, and agreement studies that use one block per tumor will overstate reproducibility.

Second, and more consequentially, because microenvironmental NRF2 activation can itself restrain the progression of NRF2-activated tumors [136], a bulk measurement that sums malignant and stromal signals may not merely be noisy but may point in the wrong direction. A tumor in which stromal NRF2 predominates and tumor-cell NRF2 is low could be scored NRF2-high and assigned to NRF2-directed therapy on the basis of a signal arising from the compartment least likely to benefit from it.

Region-resolved or single-cell measurement is therefore not a refinement of the biomarker problem but part of its definition. We regard the prospective evaluation of any NRF2-activation score as requiring, at a minimum, multi-region sampling or a spatially resolved readout, and we treat single-region immunohistochemistry as insufficient to meet the compartment-resolution requirement set out in stage (v) of the biomarker-development roadmap in Section 8.3.

Supplementary Note S2. Literature search strategy, eligibility criteria, evidence-grading rubric, and claim-level evidence map

S2.1. Search and review scope

This Supplementary Note provides the methodological detail supporting Section 1.1 of the main text. The review was conducted as a structured narrative synthesis rather than as a systematic or scoping review. Accordingly, the procedures below are reported to make literature identification and evidence appraisal transparent and auditable, but the search should not be interpreted as an exhaustive systematic search.

PubMed was the primary bibliographic database. Google Scholar was used for supplementary discovery, and ClinicalTrials.gov was consulted only to verify trial registration and status. Database searches were supplemented by backward and forward citation tracking of key primary studies. The final PubMed search was conducted on 16 September 2026.

Study identification, selection, and evidence appraisal were performed by the authors and reconciled by consensus. Duplicate independent screening and formal adjudication were not undertaken, and no protocol was prospectively registered.

Table S1. Search sources, terms, combinations, and limits.

Concept block	Search terms as executed	Notes
C1 – Pathway	NRF2 OR NFE2L2 OR "nuclear factor erythroid 2-related factor 2" OR KEAP1 OR "Kelch-like ECH-associated protein 1"	Both gene and protein designations retained because of documented transcript–protein discordance (Section 4.5)
C2 – Disease	"breast cancer" OR "breast neoplasms" OR "breast carcinoma" OR TNBC OR "triple-negative breast cancer" OR "basal-like breast cancer"	Receptor-defined and intrinsic-subtype terms both included
C3 – Redox / cell death	ferroptosis OR "lipid peroxidation" OR GPX4 OR "glutathione peroxidase 4" OR SLC7A11 OR xCT OR cystine OR "system xc" OR ACSL4 OR "oxidative stress" OR glutathione	Used with C1 and with C2
C4 – Therapy resistance	"drug resistance" OR chemoresistance OR "therapy resistance" OR radioresistance OR "endocrine resistance" OR tamoxifen OR "immune checkpoint"	Used with C1 AND C2
C5 – Biomarker / stratification	biomarker OR immunohistochemistry OR "patient stratification" OR prognosis OR "predictive marker"	Used with C1 AND C2
Combinations run	(C1 AND C2); (C1 AND C3); (C2 AND C3); (C1 AND C2 AND C4); (C1 AND C2 AND C5)	—
Sources	PubMed (NLM) as the primary database; Google Scholar for supplementary discovery; ClinicalTrials.gov for trial registration and status only	ClinicalTrials.gov was not used as a source of efficacy evidence
Supplementary methods	Backward and forward citation tracking of the primary studies anchoring each section	Applied iteratively until no further eligible primary reports were identified for a given section
Limits	Database inception to 16 September 2026; English-language reports only	Final PubMed search: 16 September 2026

S2.2. Study selection and evidence appraisal

Reports were selected against the criteria in Table S2 and appraised against the rubric in Table S3. The rubric is the one applied in Table 2 of the main text and is worded identically there, so that any grade in Table 2 can be checked against its definition here.

Table S2. Eligibility criteria as operationalized.

Domain	Included	Excluded
Topic	Mechanistic, prognostic, or therapeutic evidence concerning the NRF2–KEAP1 axis, ferroptosis, or redox metabolism with relevance to breast cancer	Reports with no identifiable link to NRF2–KEAP1 signaling, ferroptosis, or redox metabolism
Study type	Primary experimental studies (cell, organoid, animal); human tissue and cohort studies; randomized and non-randomized clinical studies; genomic atlas and cohort studies informing subtype mapping	Purely descriptive reports lacking mechanistic or clinical relevance, except cohort or atlas studies used for subtype mapping
Role of reviews	Reviews cited only to establish background consensus or to position the present synthesis against prior reviews (Table 1)	Reviews used as the primary source for a specific experimental result
Model system	Breast cancer models and human breast tumors; non-breast KEAP1/NFE2L2-driven tumors retained only where labeled as extrapolated evidence	Non-breast evidence presented without an explicit extrapolation label
Language and period	English; database inception to 16 September 2026	Non-English reports; reports appearing after the final search date
Weighting when evidence conflicted	Human tissue-level and functional evidence given greater weight	Transcript-only associations and single-model studies given lesser weight and labeled accordingly

Table S3. Evidence-grading rubric applied in Table 2 of the main text.

Item	Operational definition	What it does NOT mean
Unit of grading	Grades describe the directness and strength of the evidence available for the specific dimension being graded, not an overall judgment of the subtype.	A dimension-level grade is not a summary of the subtype; Table 2 reports the summary separately in the Overall evidence strength row.
High (H)	The dimension is supported by direct breast-cancer evidence of the type appropriate to that dimension, from a study designed to address it — a well-powered human cohort for prevalence or survival; functional manipulation in breast cancer models for mechanism — without contradictory evidence.	Does not mean independent replication where the supporting study count is one, and does not mean prospective validation of NRF2-directed patient selection.
Moderate (M)	Supported by indirect, single-model, or partially concordant evidence, or by substantial extrapolation from other tumor types.	Does not mean the association is weak in effect size; the grade describes evidence structure, not magnitude.
Low (L)	Sparse, absent, transcript-only, or wholly extrapolated evidence.	Does not mean absence of effect; absence of evidence is not evidence of absence.
Convergence criterion	Human cohort data plus protein or pathway evidence plus functional validation in breast cancer models. This criterion is applied only to the Overall evidence strength row of Table 2.	Does not apply to the individual dimensions, several of which cannot satisfy it by construction — a cell-line evidence row cannot contain cohort

Item	Operational definition	What it does NOT mean
		data, and a survival-association row cannot contain functional validation.
Study-count tags	Each evidence-feature cell of Table 2 carries a bracketed tag giving the number and type of key supporting studies, e.g. [1 retrospective TMA, n=203]; [0 direct; inferred] denotes reliance on inference from other subtypes or tumor types.	A tag of [0 direct] identifies an evidence gap, not a negative finding.
Dimensions graded	The eight feature rows of Table 2: NFE2L2/KEAP1 somatic mutation; NRF2 protein and activity; KEAP1 status; NRF2 target-gene signature; cell-line functional evidence; patient survival association; therapy-resistance link; ferroptosis relevance.	The research-priority row of Table 2 states a priority in words and is not graded, because it records an authors' judgment rather than a body of evidence. Clinical readiness and overall evidence strength are reported as separate summary rows.
Clinical readiness (separate axis)	Reported separately from evidence strength as Not validated / Exploratory / Candidate.	A High evidence grade is never to be read as clinical readiness; clinical actionability is Low across all subtypes.

S2.3. Claim-level evidence mapping

Table S4 maps each principal statement of the review to the studies supporting it, so that published evidence can be separated from the authors' own inference. Levels of inference are defined in Section 1.1 of the main text: Demonstrated, Mechanistically supported, and Proposed or testable. Reference numbers follow the main-text reference list; all cited works also appear in the main text and in its reference list. Roman numerals in the fifth column give the evidence tier defined in Section 1.1 of the main text: (i) direct breast-cancer clinical evidence; (ii) breast-cancer preclinical evidence; (iii) evidence obtained in other tumor types; (iv) a conceptual proposal of the present authors.

Table S4. Claim-level evidence map for the principal statements of the review.

Principal statement	Section	Key refs	Evidence type	Breast-native or extrapolated	Level of inference
NRF2 activation in breast cancer is predominantly non-mutational	4.1	6, 74, 75	Human genomic cohort (TCGA); pan-cancer mutation catalogue	(i) Breast-native	Demonstrated
NFE2L2 transcript is an unreliable surrogate for NRF2 pathway activity	4.5	65, 66, 76, 94, 95	Human cohorts; meta-analysis; cell studies	(i) Breast-native	Demonstrated (discordance)
Nuclear NRF2-high with cytoplasmic KEAP1-low marks a poor-prognosis TNBC subset	4.4	65	Single retrospective tissue microarray, n = 203; median H-score threshold	(i) Breast-native	Demonstrated (association only; single cohort, data-derived threshold)
MSL TNBC cells activate NRF2 by cystine-driven KEAP1 cysteinylation, with a glutathione-independent OSGIN1 arm	4.4	66, 73	Cell lines	(ii) Breast-native	Demonstrated in vitro
ACSL4 expression conditions ferroptosis sensitivity in basal-like breast cancer lines	5.2	67	Genome-wide CRISPR screen; lipidomics; cell lines	(ii) Breast-native	Demonstrated in vitro
Tamoxifen induces NRF2 in ER-positive tumors and confers cross-resistance to irradiation	6.1	64, 97, 124	Cell models plus paired patient biopsies (NCT00738777)	(i) and (ii) Breast-native	Demonstrated (human on-treatment induction)
NRF2 protein in tumor and stroma is associated with failure of pathological complete response after neoadjuvant chemotherapy	4.3, 6.2	79	Retrospective paired pre-/post-chemotherapy biopsies; HER2-positive n = 75, triple-negative n = 56; immunohistochemistry scored separately in tumor and stroma	(i) Breast-native	Demonstrated (association only; single cohort, surrogate endpoint)
NRF2-driven ferroptosis defense contributes to resistance across modalities	5.1, 6.4	70, 71, 117	Breast cells and xenografts (70, 71); hepatocellular carcinoma (117)	(ii) and (iii) Mixed	Demonstrated (breast, preclinical) + Mechanistically supported
NRF2 activation opposes checkpoint-blockade efficacy	6.5	128, 129, 130, 131	Breast preclinical (128); CD8 mechanism (129); lung adenocarcinoma (130, 131)	(iii) Chiefly extrapolated	Mechanistically supported
Host and stromal NRF2 modify breast tumor behavior in a compartment-dependent manner	4.5, 6.5	96, 132, 133, 136, 134, 135	Mouse models; spatial transcriptomics	(ii) Breast-native (96, 132, 133, 134, 135); general (136)	Demonstrated (animal)
Direct NRF2 inhibitors are not	7.3.1	140, 141, 149	Mass-spectrometry	(iii)	Demonstrated

Principal statement	Section	Key refs	Evidence type	Breast-native or extrapolated	Level of inference
clinic-ready; brusatol is not NRF2-selective			profiling; NSCLC models	Extrapolated	(pharmacology)
Glutaminase inhibition exploits a KEAP1-loss metabolic dependency	8.2	143, 144	Lung adenocarcinoma; KEAPSAKE trial terminated early	(iii) Extrapolated	Mechanistically supported; cautionary
A candidate redox-addicted TNBC subset defined by nuclear NRF2-high, KEAP1-low, SLC7A11/cystine reliance, ACSL4 lipid program and GPX4 ferroptosis defense	4.4	65, 66, 67, 73	Assembled from different datasets and experimental systems	(iv) Breast-native components; composite untested	Proposed / testable — co-occurrence not demonstrated; prevalence unknown
Composite NRF2-activation score (Box 1)	8.3	153 (precedent, non-breast)	Conceptual construct	(iv) Not applicable	Proposed / testable — not analytically or clinically validated
Predictions P1–P5	8.4	—	Falsifiable predictions derived from the framework	(iv) Not applicable	Proposed / testable
